# Macrophages induce AKT/β-catenin-dependent Lgr5^+^ stem cell activation and hair follicle regeneration through TNF

**DOI:** 10.1038/ncomms14091

**Published:** 2017-03-27

**Authors:** Xusheng Wang, Haiyan Chen, Ruiyun Tian, Yiling Zhang, Marina S. Drutskaya, Chengmei Wang, Jianfeng Ge, Zhimeng Fan, Deqiang Kong, Xiaoxiao Wang, Ting Cai, Ying Zhou, Jingwen Wang, Jinmei Wang, Shan Wang, Zhihai Qin, Huanhuan Jia, Yue Wu, Jia Liu, Sergei A. Nedospasov, Edward E. Tredget, Mei Lin, Jianjun Liu, Yuyang Jiang, Yaojiong Wu

**Affiliations:** 1 School of Life Sciences, Tsinghua University, Beijing 100084, China; 2 The Shenzhen Key Laboratory of Health Sciences and Technology, Graduate School at Shenzhen, Tsinghua University, Shenzhen 518055, China; 3 Tsinghua-Berkeley Shenzhen Institute (TBSI), Tsinghua University, Shenzhen 518055, China; 4 State Key Laboratory Breeding Base-Shenzhen Key Laboratory of Chemical Biology, Graduate School at Shenzhen, Tsinghua University, 518055 Shenzhen, China; 5 Shenzhen Peiyuan Biotechnology Company, Shenzhen 518055, China; 6 Department of Orthopedics, The General Hospital of Chinese People's Liberation Army, Beijing 100039, China; 7 Engelhardt Institute of Molecular Biology, Russian Academy of Sciences, Moscow 119991, Russia; 8 Institute of Biophysics, Chinese Academy of Sciences, Beijing 100101, China; 9 Guangdong Laboratory Animals Monitoring Institute, Guangdong Provincial Key Laboratory of Laboratory Animals, Guangzhou 510260, China; 10 College of Life Sciences, Northeast Forestry University, Harbin 100040, China; 11 Wound Healing Research Group, Department of Surgery, University of Alberta, Edmonton, Alberta, Canada ABT6G2E1; 12 Medical Key Laboratory of Health Toxicology of Shenzhen, Shenzhen Center for Disease Control and Prevention, Shenzhen 518054, China

## Abstract

Skin stem cells can regenerate epidermal appendages; however, hair follicles (HF) lost as a result of injury are barely regenerated. Here we show that macrophages in wounds activate HF stem cells, leading to telogen–anagen transition (TAT) around the wound and *de novo* HF regeneration, mostly through TNF signalling. Both TNF knockout and overexpression attenuate HF neogenesis in wounds, suggesting dose-dependent induction of HF neogenesis by TNF, which is consistent with TNF-induced AKT signalling in epidermal stem cells *in vitro*. TNF-induced β-catenin accumulation is dependent on AKT but not Wnt signalling. Inhibition of PI3K/AKT blocks depilation-induced HF TAT. Notably, *Pten* loss in Lgr5^+^ HF stem cells results in HF TAT independent of injury and promotes HF neogenesis after wounding. Thus, our results suggest that macrophage-TNF-induced AKT/β-catenin signalling in Lgr5^+^ HF stem cells has a crucial role in promoting HF cycling and neogenesis after wounding.

## Abstract (web summary)

Hair can be regenerated after skin wounding. Here the authors show that inflammatory macrophages produce TNF that activates Wnt signalling in hair follicle stem cells to drive this hair regeneration after wound repair in mice.

Hair follicles are complicated anatomical structures that harbour stem cells capable of regenerating the epidermis and its appendages^1^,^2^,^3^,^4^,^5^. Wound-induced hair anagen re-entry/growth (WIH-A) occurs in tissue adjacent to the wound site^6^, and ablation of macrophages, especially during an early phase of wound healing, inhibits wound repair^7^,^8^,^9^. In Salamander, macrophages are required for limb regeneration after amputation^10^, suggesting that macrophage-derived molecules create a regenerative environment in the injured tissue. Apoptosis signal-regulating kinase 1-dependent recruitment and activation of macrophages is essential for wound-induced hair growth^11^, and macrophages activate stem cells in hair follicle (HF) cycling^12^,^13^. In hair plucking shin injury, tumour necrosis factor (TNF)-secreting macrophages contribute to the cooperative activation of HF stem cells^14^. After tissue injury, all macrophages (Ly6C^+^ inflammatory macrophages and CX3CR1^+^ tissue-resident macrophages) are activated and differentiate into M1-like inflammatory cells, and migrate into the wound site^15^,^16^,^17^,^18^,^19^, but the distinct role of macrophage subpopulations in HF stem cell activation is unclear. In addition, a systematic analysis of cytokine release by different immune cells in the early phase of wound healing is needed to provide a profile of immune cell mediators (especially macrophages) that interact with HF stem cells. WIH follicle neogenesis (WIHN) is an example of embryonic-like regeneration, a type of regenerative response that is rare in mammals^20^,^21^,^22^. γδ T cells from dermis are known to be important for HF neogenesis after wounding^23^, but the role of macrophages in HF neogenesis after wounding is unexplored. In addition to macrophage heterogeneity, HF stem cells are composed of different stem cell subpopulations^3^,^4^,^5^,^24^, among which Lgr5^+^ cells are the first stem cells activated during telogen–anagen transition in hair cycle. These cells substantially contribute to the cycling of anagen HFs, and Lgr5^+^ progeny repopulate other stem cell compartments in the HF^4^. However, the role of Lgr5^+^ cells in wound-induced hair growth and neogenesis is still mostly unknown. Wnt signalling is important for HF morphogenesis at the embryonic stage and hair cycle after birth^25^,^26^,^27^,^28^, and also important for WIHN^22^,^23^,^28^; however, the underlying mechanisms that regulate Wnt signalling are unclear.

Here, we evaluate the role of Ly6C^+^ inflammatory macrophages and CX3CR1^+^ tissue-resident macrophages in WIH-A and WIHN, via ablation of either of these two macrophage populations in genetic mouse models. And the result shows that Ly6C^+^ inflammatory macrophages are required for WIH-A and WIHN. To systematically analyse influence of Ly6C^+^ and CX3CR1^+^ macrophages upon the HF stem cells, microarray was performed to detect the differently expressed genes in two macrophage sub-populations, combined with gene deficient mouse models, we show that TNF has a crucial role in both WIH-A and WIHN. To understand the TNF-induced signal changes in epithelial HF stem cells, we performed iTRAQ analysis; the result shows TNF induces AKT phosphorylation (p-AKT) in epidermal stem cells (Epi-SCs) and Lgr5^+^ cells, and AKT signals positively regulate β-catenin signalling. To further explore the role of AKT signalling in WIH-A and WIHN, we developed both Lgr5+ cells deletion and *Pten* knockout mice model, and the result shows that Lgr5^+^ HF stem cells have an important function in both WIH-A and WIHN, and elevated AKT (*Pten* loss) signalling in Lgr5^+^ HF stem cells promotes WIHN.

## Results

### Ly6C^+^ macrophages are required for WIH-A and WIHN

**Figure 1:**
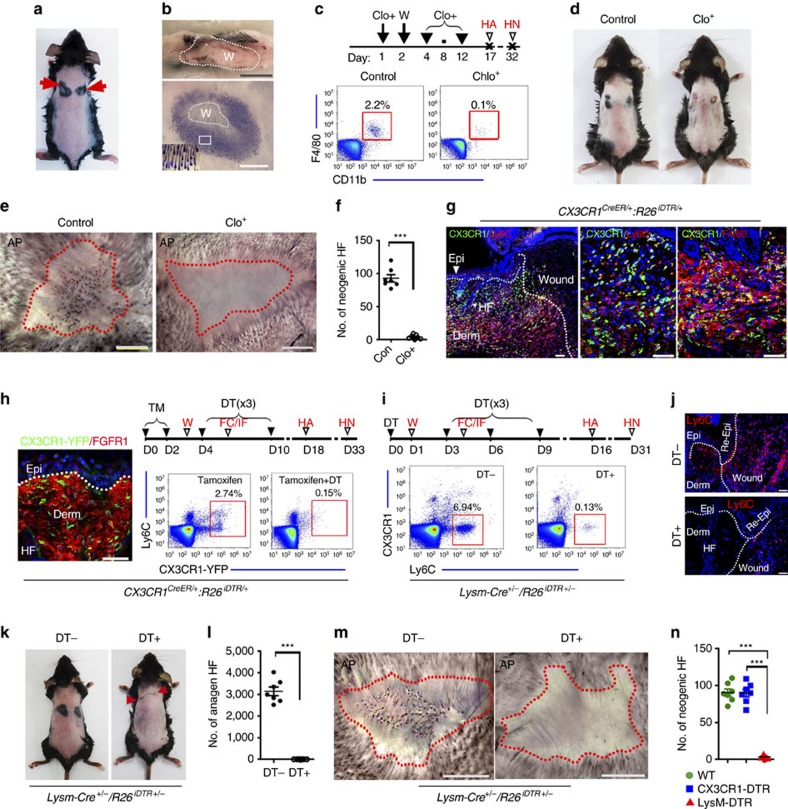
Ly6C^+^ inflammatory macrophages contribute to WIH-A and WIHN. (**a**) Wounding the skin of 8-week-old mice induced a transition of HF from the telogen phase to the anagen phase around wound areas. *n*=56. (**b**) The longitudinal section of the wound at the 15th day post-wound (PWD-15) (upper panel) and of the dermal side of the skin showed the pigmented anagen HFs around the wound (lower panel). (**c**) Flow cytometry analysis of wound tissue single cell suspensions showed the extensive depletion of F4/80^+^/CD11b^+^ macrophages after clodronate liposome (Clo) treatment. Mice receiving clodronate, *n*=7; control mice, *n*=12. (**d**) WIH-A analysis in Clo treated (*n*=8) and the control mice (*n*=11). (**e**,**f**) Clo treatment resulted in complete elimination of WIHN (*n*=7) (**e**), and the number of neogenic HFs in the two groups of mice was quantified (**f**). (**g**) Immunofluorescence (IF) analysis of wound tissue showed the infiltration of both Ly6C^+^ inflammatory macrophages and CX3CR1^+^ resident macrophages at PWD-3. For all IF analyses, representative images from 8 to 16 tissue sections of wounds in 4–6 mice are shown. (**h**) IF image showed CX3CR1-YFP cells (green) in the dermis of the normal skin of *CX3CR1*^*CreER*/+^*:R26*^*iDTR*/+^ mice. Flow cytometry analysis indicated that TM and DT treatments depleted over 90% of CX3CR1-EYFP cells in the blood of *CX3CR1*^*CreER*/+^*:R26*^*iDTR*/+^ mice (*n*=9). (**i**) Flow cytometry analysis showed that the DT treatments depleted over 95% of Ly6C^+^ cells in the blood of *LysM-Cre:R26*^iDTR/+^ mice (*n*=9). (**j**) IF staining of Ly6C^+^ macrophages in the wound tissue (PWD-3) of *LysM-Cre:R26*^*iDTR/+*^ mice with or without DT treatment. (**k**,**l**) Treatment of *LysM-Cre:R26*^*iDTR/+*^ mice with DT resulted in the complete inhibition of WIH-A. *n*=8 for DT- mice; *n*=10 for DT^+^ mice. (**m**,**n**) Deletion of Ly6C^+^ macrophages resulted in no neogenic HF in the wound (*n*=7), but the deletion of CX3CR1^+^ macrophages showed no obvious effect on the number of neogenic HF (*n*=8 mice). W, wound; HA, hair follicle anagen analysis; HN, hair follicle neogenesis analysis; TM, tamoxifen; DT, diphtheria toxin; WT, wild type. Scale bars, 50 μm. Data are expressed as the mean±s.e.m. ***P<0.005, unpaired *t*-test, two-tailed.

We investigated the mechanism underlying WIH-A, in which wounding of the skin induced the transition from telogen HFs (postnatal days P46–70) to anagen HFs near the wound in 8-week-old (P60–70) C57/B6 mice (an excisional wound 5 mm in diameter generally induces 2,000–4,000 anagen HFs adjacent the wound) (Fig. 1a,b; Supplementary Fig. 1a,b). First, we evaluated the involvement of inflammation in WIH-A. The systemic administration of dexamethasone or JSH-23 (an NF-κB inhibitor)^29^,^30^, which both suppress inflammatory cell infiltration, completely abolished WIH-A (Supplementary Fig. 1c–h). To examine the contribution of macrophages, we depleted macrophages with clodronate liposomes, which reduced macrophages to barely detectable levels in the wound (Fig. 1c). Intriguingly, successive clodronate treatment completely abolished WIH-A and was associated with delayed wound healing (Fig. 1d). In addition, macrophage depletion led to the loss of WIHN (Fig. 1e,f).

We next examined the contribution of different macrophage subsets to WIH-A and WIHN. Macrophages are classified into two distinct subsets based on their origin^16^,^18^,^31^,^32^, and both Ly6C^+^ inflammatory macrophages and CX3CR1^+^ tissue-resident macrophages abundantly infiltrated into wounds (on the third day post-wounding, PWD-3) (Fig. 1g). To verify that both macrophage sub-populations contributed to WIH-A and WIHN in different ways, we depleted each sub-population individually. First, tissue-resident macrophages were selectively depleted in *CX3CR1*^*CreER/+*^*:R26*^*iDTR/+*^ mice. CX3CR1-EYFP^+^ cells, which were also positive for F4/80 and largely for MHC II^+^ (Supplementary Fig. 2a), were detected in the normal skin dermis (Fig. 1h). After treatment with tamoxifen (TM) and diphtheria toxin (DT), over 90% of CX3CR1-EYFP cells were depleted in the blood, skin (Fig. 1h; Supplementary Fig. 2b), and skin wound sites at PWD-3 (Supplementary Fig. 3a). CX3CR1^+^ macrophage depletion did not affect WIH-A (Supplementary Fig. 3e). We then analysed the influence of myeloid-derived Ly6C^+^ macrophages on WIH-A. The administration of DT in *LysM-Cre: R26*^*iDTR/+*^ mice specifically depletes myeloid-derived macrophages^33^. Repetitive injections of DT (heterozygous, 25 ng g^−1^ body weight) largely eliminated Ly6C^+^ macrophages in the blood and in the wounds (Fig. 1i,j) and completely abolished WIH-A (Fig. 1k,l) and WIHN (Fig. 1m,n) in mice. Conversely, CX3CR1^+^ macrophage depletion in mice resulted in negligible effects on both WIH-A (Supplementary Fig. 3e,f) and WIHN (Fig. 1n). The expression of CCL2, the major ligand of CCR2 (CCR2 is crucial for the recruitment of Ly6C^+^ macrophages into wounds^32^,^34^), was upregulated in wound-adjacent tissue, and the kinetics of *CCL2* expression at different time points showed peak at PWD-3. Consistently, immunostaining analysis showed CCL2 was detected in the hair germ of unwounded skin, and was detected in the hair germ, HF infundibulum and some epidermal cells and dermal cells at PWD-1. At PWD-3.5, the levels of CCL2 increased in dermal cells but decreased in the hair germ and infundibulum (Supplementary Fig. 3b,c). This finding was further validated by treatment with a CCR2 antagonist or CCR2 siRNA; both markedly reduced the number of Ly6C^+^ macrophages in the wound (Supplementary Fig. 3d) and dramatically attenuated WIH-A (Supplementary Fig. 3e,f). Together, these results indicate that Ly6C^+^ inflammatory macrophages, but not CX3CR1^+^ tissue-resident macrophages, are responsible for both WIH-A and WIHN.

### TNF is a crucial mediator of macrophage-induced HF TAT

**Figure 2:**
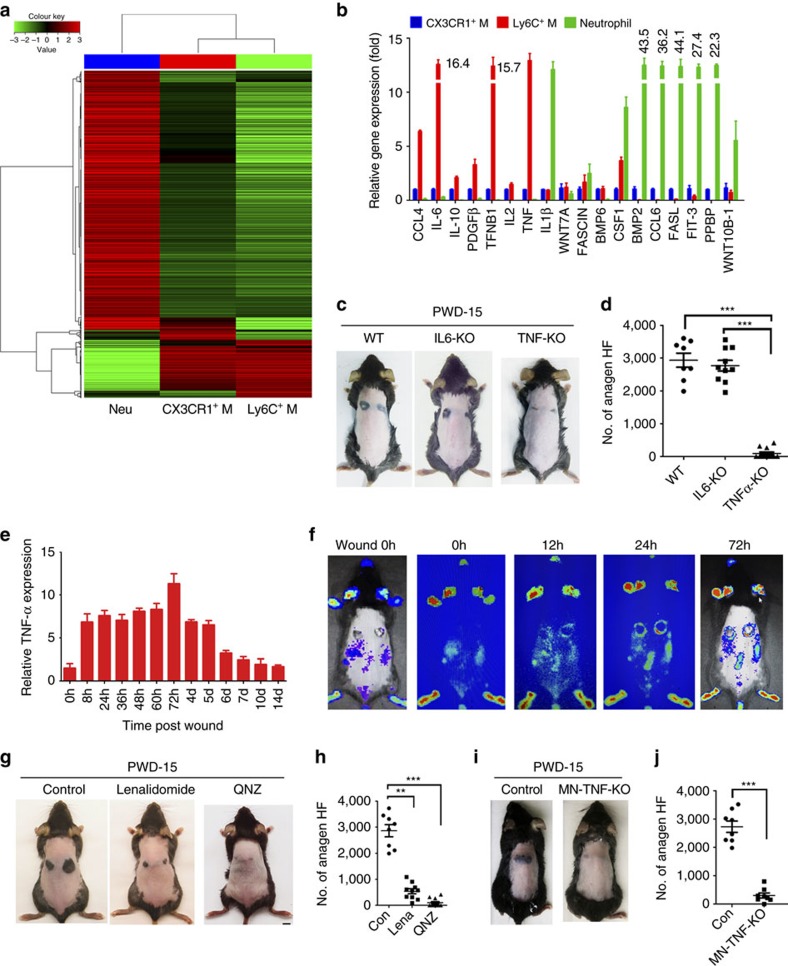
TNF is a crucial mediator to induce the HF TAT. (**a**) Gene expression profile of Ly6C^+^/F4/80^+^ macrophages (M), CX3CR1^+^/F4/80^+^ macrophages and Ly6G^+^/F4/80^−^ neutrophils (Neu), which were sorted from wound tissue at PWD-3. Total RNA was extracted from samples of three mice. (**b**) Differentially expressed cytokine genes from microarray analysis were further validated by real-time PCR analysis. (For all real-time PCR analyses, gene expression was normalized to GAPDH with 40 cycles, data are represented as the mean±s.d., and *n*=3.) (**c**,**d**) WIH-A analysis in WT and deficient for *IL6* (IL6-KO) or *TNFA* (TNFα-KO) gene mice, and the number of anagen HFs in different mice was quantified (**d**). *IL6*-knockout mice, *n*=5; *TNFA*-knockout mice, *n*=6; WT mice, *n*=6. (**e**) Real-time PCR analysis showed *TNFA* expression levels in the tissue surrounding the wounds (2 mm in width) at different times post-wounding. (**f**) Bioluminescent imaging of *Tnf-Luc-eGFP* mice at different times after wounding showed changes of the TNF-α level in the wound. Data are representative of 7–9 independent experiments at each time point. (**g**,**h**) Lenalidomide and QNZ treatment resulted in decreased anagen HFs as assessed at PWD-15 (**g**), and the number of anagen HFs in different groups was quantified (**h**). Control mice, *n*=9; lenalidomide-treated mice, *n*=7; QNZ-treated mice, *n*=8. (**i**,**j**) Wound-induced anagen HFs was significantly decreased in *Lysm*^*cre/+*^*:TNF*^*flox/flox*^ mice (**i**), and the number of anagen HFs in two groups was quantified (**j**). Control mice (*Lysm*^*cre/+*^*:TNF*^*flox/+*^), *n*=6; *Lysm*^*cre/+*^*:TNF*^*flox/flox*^ mice, *n*=4. Data are expressed as the mean±s.e.m. **P<0.01, ***P<0.005, unpaired *t*-test, two-tailed.

To identify mediators of macrophage-induced HF TAT, we performed a microarray analysis of Ly6C^+^ inflammatory macrophages (Ly6C^+^/F4/80^+^), CX3CR1^+^ (CX3CR1^+^/F4/80^+^) resident macrophages and neutrophils (Ly6G^+^/F4/80^−^) that were isolated from wound tissues at PWD-3. The results revealed a disparity of gene expression among these cells: Ly6C^+^ macrophages expressed higher (≥5-fold) levels of 241 genes compared with those in CX3CR1^+^ macrophages and higher levels of 3382 genes compared with those in neutrophils (Fig. 2a). The differentially expressed genes in Ly6C^+^ macrophages and CX3CR1^+^ macrophages are presented in Supplementary Table 1. Real-time PCR analysis confirmed the differential expression of cytokine genes; Ly6C^+^ macrophages expressed greater amounts of *CCL4* (6.3 × ), *IL6* (16.4 × ), *IL10* (2.2 × ), *PDGFB* (3.3 × ) and *TNFA* (12.8 × ) and lower levels of *CSF1, BMP2, CCL6, FASL, FLT3, PPBP* and *WNT10B* (Fig. 2b). To validate the involvement of interleukin (IL)-6 and TNF in WIH-A, we performed a functional analysis using gene-deficient mice. Wounding induced similar amounts of anagen HFs in mice that were deficient for *IL6* compared with those seen in wild-type mice; in contrast, wounding of *TNFA*^*−/−*^ mice failed to induce anagen HFs at PWD-15 (Fig. 2c).

Wounding induced a marked upregulation of the *TNFA* gene in the surrounding tissue, which peaked at 72 h after initiation of the wound and returned to basal levels after PWD-7 (Fig. 2e). Consistent with this result, a time course of TNF expression after wounding in *Tnf-Luc-eGFP* reporter mice exhibited a similar pattern of up-regulation; in addition, tissue areas with elevated TNF matched the areas that subsequently underwent HF TAT (Fig. 2f). Based on further analysis, TNF in the wound was primarily derived from macrophages and largely co-localized to Ly6C^+^ macrophages (Supplementary Fig. 4a–c). The effects of TNF on WIH-A were further verified by inhibiting TNF in macrophages using lenalidomide (which inhibits TNF secretion) or QNZ (EVP4593, which inhibits TNF production); treatment with either drug led to marked reductions in the number of wound-induced anagen HFs, particularly QNZ (Fig. 2g,h). To further verify the contribution of macrophage-derived TNF, we employed *Lysm*^*cre/+*^*:TNF*^*flox/flox*^ mice^35^, in which macrophages and neutrophils do not express *TNFA*. Wounding was associated with a significantly (*P*<0.005) lower number of anagen HFs in these mice compared with the numbers seen in their littermate controls (Fig. 2i,j). Moreover, subcutaneous injection of lipopolysaccharide-treated macrophages induced HF TAT at the injection site^11^, while the injection of *TNFA*^*−/−*^ macrophages failed to induce HF TAT (Supplementary Fig. 4d).

### TNF is sufficient to induce HF TAT and is crucial for WIHN

**Figure 3:**
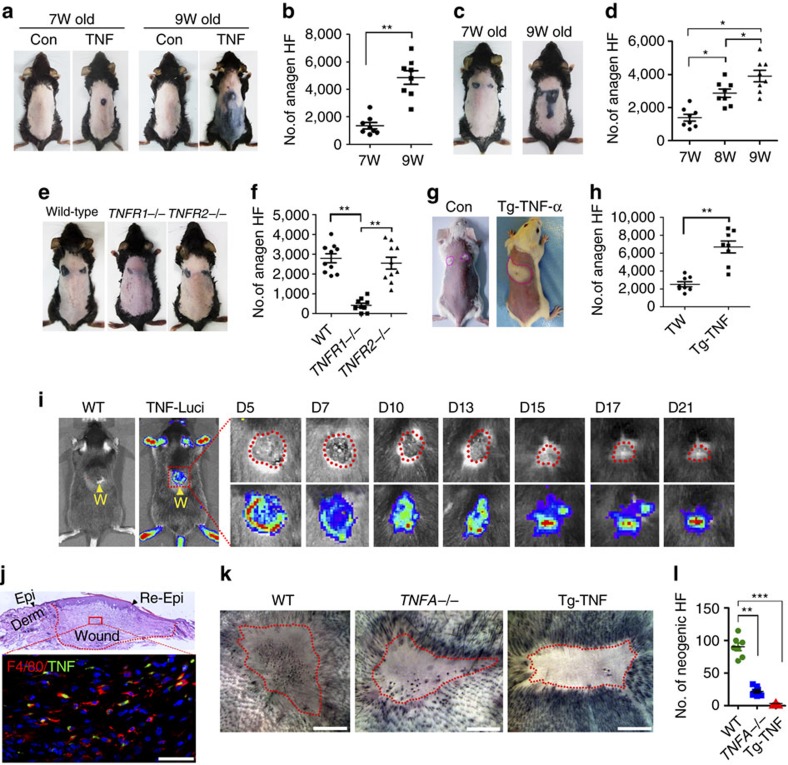
TNF-α is sufficient to induce HF TAT and is crucial for WIHN. (**a**,**b**) Intracutaneous injection of TNF-α can induce the HF telogen–anagen transition at the injection site in 7-week (W)-old mice (refractory phase) and 9-week-old mice (competent phase) (**a**), and the number of TNF-α-induced anagen hair follicles in the two different group was quantified (**b**). Seven-week-old mice, *n*=6 for each group; 9-week-old mice, *n*=8 for each group. (**c**,**d**) Wounding to the skin induced more anagen HFs in 9-week-old mice than in 7-week-old mice (**c**), and the number of anagen HFs in the two different groups was quantified (**d**). *n*=7 for each age group. (**e**,**f**) WIH-A analysis in *TNFR1*^−/−^ mice, *TNFR2*^*−/−*^ mice and WT mice (**e**), and the number of anagen HFs in different groups was quantified (**f**). Wild-type (C57BL/6) mice, *n* =7; *TNFR1*^−/−^ mice, *n*=6; *TNFR2*^−/−^ mice, *n*=9. (**g**,**h**) Wound-induced anagen HFs in mice constitutively expressing TNF-α (Tg-TNF-α) were significantly increased compared with WT mice, and the number of anagen HFs in the two different groups was quantified (**h**). Control mice (WT), *n*=6; Tg-TNF-α mice, *n*=5. (**i**) Bioluminescent imaging of *Tnf-Luc-eGFP* mice at different days (D) after wounding showed changes in the TNF-α level in the wound. Areas with high TNF-α levels (green/red) shifted from the wound (W) periphery to the wound centre with the progression of wound healing. *n*=15 for *Tnf-Luc-eGFP* mice, and *n*=10 for wild-type (WT) mice. (**j**) IF analysis of sections from the PWD-14 wound showed the presence of F4/80^+^ macrophages in the wound, which largely co-localized with TNF-α. Scale bar, 50 μm. (**k**,**l**) WIHN analysis in WT, *TNFA*^*−/−*^ and Tg-TNF-α mice at PWD-30 (**k**), and the number of neogenic HFs in wounds was quantified (**l**). *n*=6 for both wild-type and *TNFA*^*−/−*^ mice; *n*=12 for Tg-TNF-α mice. Scale bars, 2 mm. Data are expressed as the mean±s.e.m. *P<0.05, **P<0.01, ***P<0.005, unpaired *t*-test, two-tailed.

To gain further insight into the role of TNF in HF stem cells activation and HF regeneration, we first examined whether a gain of TNF was sufficient to induce HF TAT independent of wounding. The subcutaneous injection of TNF (200 ng of recombinant mouse TNF-α in 50 μl of Matrigel) induced HF TAT in both refractory (7-week-old) and competent telogen (9-week-old) mice at the injection site, with more TAT HFs observed in the 9-week-old mice (∼5,000 anagen HFs) than in the 7-week-old mice (<2,000 anagen HFs) (Fig. 3a,b), consistent with the findings of a recent study^14^. Consistently, wounding induced more anagen HFs in 9-week-old mice than in 7-week-old mice (Fig. 3c,d). TNF potentially binds to two receptors (TNFR1 and TNFR2). Immunofluorescence analysis indicated both receptors were expressed in epidermal cells and HF cells (Supplementary Fig. 4e). Interestingly, anagen HFs were barely induced in wounded *TNFR1*^−/−^ mice, while the wounding of *TNFR2*^−/−^ mice induced similar levels of anagen HFs as wild-type mice (Fig. 3e,f), indicating TNF activates HFs largely through TNFR1. Impressively, the wounding of transgenic (Tg)-TNF mice, which constitutively overexpress TNF, induced many more anagen HFs (Fig. 3g,h). In addition, real-time PCR analysis indicated that TNF mRNA expression levels in wound-adjacent tissue (PWD-3) in Tg-TNF mice were approximately threefold higher than those in non-wounded skin (Supplementary Fig. 4f).

We next examined whether TNF is involved in *de novo* HF regeneration. TNF expression after wounding in *Tnf-Luc-eGFP* mice shifted from intense expression in the wound periphery to the centre of the wound at approximately PWD-10 (Fig. 3i). Meanwhile, substantial numbers of macrophages, the majority of which co-localized with TNF, were detected in the wound bed tissue at PWD-14 (Fig. 3j), which indicated the persistent presence of macrophages and TNF in the wound. The role of TNF in *de novo* HF regeneration was further determined by employing *TNFA*^*−/−*^ mice. Full-thickness excisional wounds were made in 3–4-week-old mice (P23 days). At PWD-30, much fewer neogenic HFs were present in *TNFA*^*−/−*^ mice compared with wild-type mice (22±11 versus 94±23, *P*<0.05). Unexpectedly, few neogenic hairs were detected in the wounds of Tg-TNF mice at the same age (Fig. 3k,l). Based on these results, TNF is necessary for *de novo* HF regeneration, but excessive TNF inhibits regeneration, suggesting that a more complicated mechanism underlies the role of TNF in HF neogenesis.

### TNF activates the AKT/β-catenin signalling in HF stem cells

**Figure 4:**
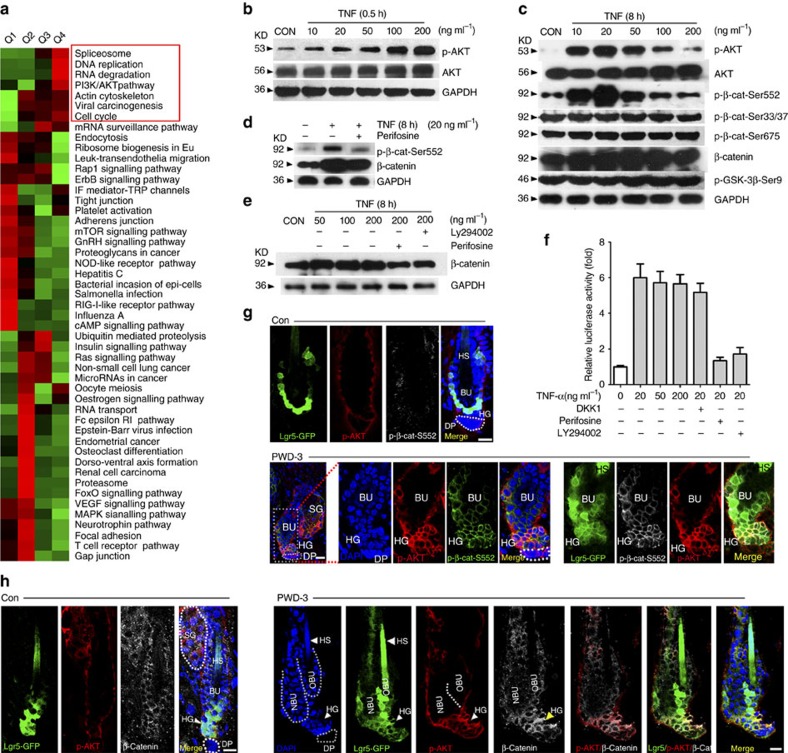
TNF activates the PI3K/AKT pathway in HF stem cells. (**a**) Using TMT labelling and affinity enrichment followed by high-resolution LC–MS/MS and quantitative phosphor-proteomics, functional enrichment-based cluster analysis identified up-regulated signals in cultured epidermal stem cells after TNF-α treatment. (**b**) Western blot analysis of p-AKT (at Serine 473) and total AKT in cultured murine epidermal stem cells in the presence of different concentrations of TNF-α for 0.5 h. For all western blot analysis, data are representative of 3–5 independent experiments. (**c**) TNF-α treatment (8 h) resulted in no obvious changes in p-GSK-3β(ser9) and p-β-catenin (Ser33/37/Thr45, Ser675) but significantly increased the level of p-β-catenin (Ser552), which also showed in a TNF-dose-dependent manner, quite similar to p-AKT. (**d**) Perifosine diminished p-β-catenin (Ser552) levels that were elevated by TNF-α in cultured epidermal stem cells. (**e**) Western blot analysis indicated that TNF-α increased the level of β-catenin, and the effect was attenuated by Perifosine or LY294002. (**f**) TCF/LEF Dual-luciferase reporter analysis showed the relative transcription activity in differently treated groups. (**g**) At PWD-3, both p-AKT (Ser473) and p-β-catenin (Ser552) were detected in the wound adjacent to Lgr5^+^ hair follicle stem cells, and the p-AKT (Ser473) and p-β-catenin (Ser552) were highly co-localized. (**h**) At PWD-3, accumulated β-catenin was detected in the wound adjacent to Lgr5^+^ hair follicle stem cells, and the accumulated β-catenin was highly co-localized with p-AKT (Ser473). Scale bars, 30 μm.

To elucidate the molecular mechanism by which TNF activates HF stem cells, we performed proteomic quantification of phosphorylation in TNF-treated and untreated Epi-SCs. After TNF (recombinant mouse TNF-α) treatment, phosphorylation levels increased (>1.5-fold) at 168 sites and decreased (<1.5-fold) at 186 sites. Bioinformatic analysis was then carried out to annotate these quantifiable targets. The most significantly upregulated phosphorylation sites were enriched for pathways related to DNA replication and the cell cycle, including the PI3K/AKT/mTOR pathway (Fig. 4a). To examine the involvement of PI3K/AKT signalling in HF stem cells activation and HF TAT, we first determined the phosphorylated AKT levels (p-AKT, Ser-473) in the skin after wounding. p-AKT was detected in integrin-α6^hi^ Epi-SCs 6 h post-wounding (Supplementary Fig. 5a). Likewise, we detected the presence of p-AKT and Ki67 in a fraction of Lgr5^+^ HF stem cells at 1.5 days after wounding, and the number of p-AKT^+^ and Ki67^+^ HF stem cells further increased after PWD-3 (Supplementary Fig. 5b).

To determine whether AKT signalling is required for HF TAT, we examined the effects of loss or gain of AKT activity. In mice, hair depilation in telogen-phase HFs (P50–80) induced HF TAT synchronously. The topical application of perifosine, a potent AKT inhibitor^36^, or LY294002 (a PI3K inhibitor) markedly suppressed hair depilation-induced TAT (Supplementary Fig. 5c,d). Conversely, the local injection of bpV (phen), a PTEN inhibitor, into the skin of mice (P50–60) that did not undergo depilation caused the marked upregulation of HF TAT at the injection site (Supplementary Fig. 5e).

To gain insight into the mechanisms underlying TNF-induced AKT signalling, we performed an *in vitro* analysis of Epi-SCs. The treatment of cultured Epi-SCs with TNF for 0.5 h markedly increased the p-AKT levels in a dose-dependent manner (Fig. 4b). Intriguingly, the treatment of Epi-SCs with TNF for a longer period of time (8 h) resulted in decreased levels of p-AKT with increased concentration of TNF. Further analysis indicated TNF treatment resulted in no obvious changes in the level of p-GSK-3β (ser9) and p-β-catenin (Ser33/37/Thr45, Ser675) but significantly increased the level of p-β-catenin (Ser552) in parallel with p-AKT levels (Fig. 4c). In addition, perifosine eliminated the TNF-induced elevation in p-β-catenin (Ser552) levels (Fig. 4d), suggesting that TNF-induced p-β-catenin (Ser552) activation is mediated by AKT signalling. Consequently, treatment with TNF (8 h) increased β-catenin levels in a PI3K/AKT-dependent manner (Fig. 4e). Furthermore, although TNF increased the expression levels of Wnt3a, Wnt7b and Wnt10b in cultured Epi-SCs (Supplementary Fig. 6a), DKK1 (a Wnt antagonist) treatment failed to reduce TNF-induced β-catenin transcriptional activity, although PI3K/AKT inhibitors did reduce transcriptional activity, as determined by the results of a TCF/LEF dual luciferase reporter assay (Fig. 4f). Taken together, our results suggest TNF activates β-catenin through the AKT-mediated phosphorylation of β-catenin (Ser552) rather than via Wnt upregulation. Moreover, immunofluorescence analysis of wound-adjacent HFs (PWD-3) indicated that p-β-catenin (Ser552) and accumulated β-catenin were highly co-localized with p-AKT signals in Lgr5^+^ HF stem cells (Fig. 4g,h).

### Lgr5^+^ HF stem cells are important for WIH-A and WIHN

**Figure 5:**
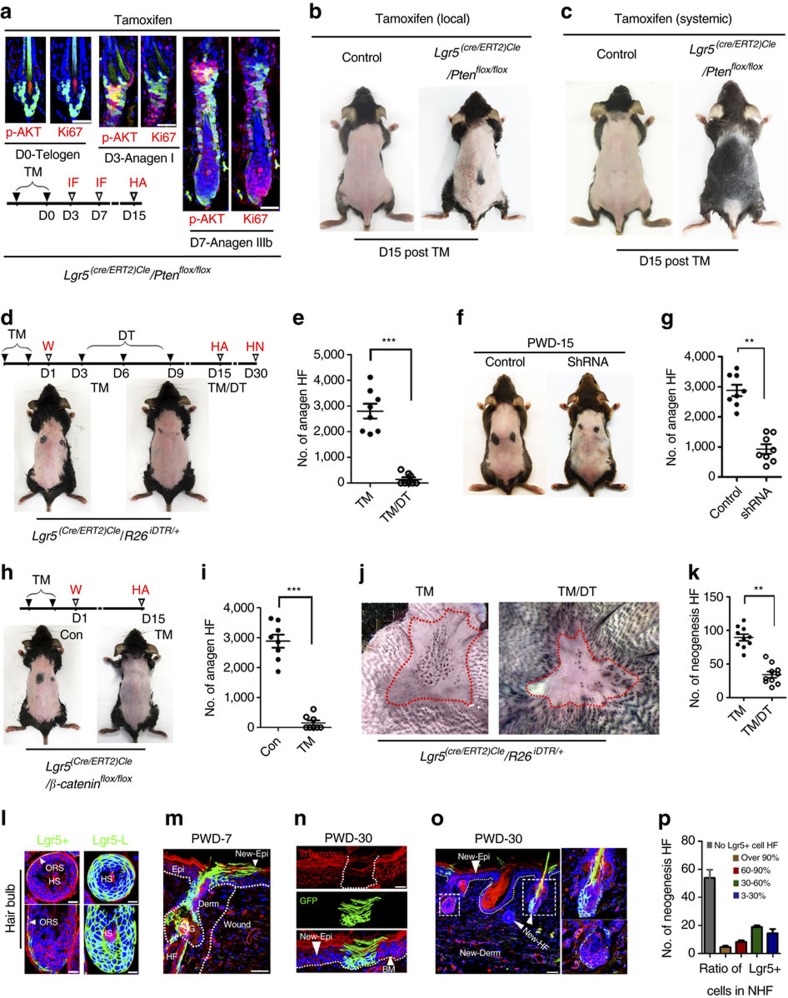
Lgr5^+^ HFSCs are indispensable for WIH-A and important for WIHN. (**a**) IF analysis of skin tissue sections indicated that tamoxifen treatment for *Lgr5-Cre:Pten*^*flox/flox*^ mice induced marked p-AKT and the division of Lgr5-EGFP^+^ cells in the HF. Scale bars, 50 μm. (**b**) Subcutaneous injection of tamoxifen induced HF TAT at the injection site in *Lgr5-Cre:Pten*^*flox/flox*^ mice (*n*=6). (**c**) Intraperitoneal administration of tamoxifen induced widespread HF TAT in back skin. *Lgr5-Cre:Pten*^flox/flox^ mice, *n*=9; WT mice, *n*=12. (**d**,**e**) Deletion of Lgr5^+^ cells in *Lgr5-Cre:R26*^*iDTR/+*^ mice completely attenuated the WIH-A (**d**), and the number of anagen HFs was quantified (*n*=7) (**e**). (**f**,**g**) Wound-induced anagen HFs were markedly decreased in mice that received *Lgr5-mTNFR1-ShRNA* (ShRNA) compared with mice that received a mock sequence. (**h**,**i**) WIH-A analysis in β-catenin knockout and the control mice at PWD-15 (*n*=7). (**j**,**k**) Depletion of Lgr5^+^ stem cells result in extensive reduction of neogenic HFs. *n*=5 for TM-treated mice, and *n*=6 for TM- and DT-treated mice. (**l**) Lgr5^+^ cells (mTomato^+^/mGFP^+^, mT^+^/mG^+^) were mainly located in the ORS of the anagen hair follicle (Anagen V), and the Lgr5^+^ cell lineage cells (mT^−^/mG^+^) contributed to all the cellular components of the hair bulb. Lgr5+, Lgr5 expressing cells. Lgr5-L, Lgr5^+^ cell lineage tracing. Scale bars, 20 μm. (**m**) Lgr5^+^ progeny cells (mT^−^/mG^+^) migrated from the HF toward the wound area at PWD-7. (**n**) Lgr5^+^ progeny cells (mT^−^/mG^+^) formed clones in newly formed epithelium at PWD-30. (**o**,**p**) Lgr5^+^ progeny cells participated in the neogenic HF in wounds (**o**), and the number of Lgr5^+^ progeny cells in different neogenic HFs varied greatly as follows: the neogenic HFs without or with less than 3% of Lgr5^+^ lineage cells comprised 53% of all analysed neogenic HFs (50 in 94), and those with 3–30%, 30–60%, 60–90% and over 90% of Lgr5^+^ lineage cells comprised 16% (15 in 94), 19% (18 in 94), 8% (7 in 94) and 4% (4 in 94) of all analysed neogenic HFs, respectively (**p**). All 94 neogenic HFs were analysed from 5 different Lgr5^+^ cell lineage tracing mice. Scale bars, 50 μm. Data are expressed as the mean±s.e.m. **P<0.01, ***P<0.005, unpaired *t*-test, two-tailed.

To further verify the role of AKT signalling in Lgr5^+^ cells to initiate anagen HFs, we generated *Lgr5-Cre(Lgr5-GFP-Cre-ERT2):Pten*^*flox/flox*^ mice. Notably, TM treatment markedly induced p-AKT activity and the division of Lgr5^+^ cells (Fig. 5a). Moreover, the intracutaneous injection (local treatment) of TM induced HF TAT (second telogen, 8–9-week-old mice) at the injection site (Fig. 5b), and the intraperitoneal administration (systemic treatment) of TM induced widespread HF TAT in the back skin of these mice (Fig. 5c), indicating that *Pten* knockout in Lgr5^+^ cells at second telogen could make the second telogen shorter. Thus, activation of the AKT pathway in Lgr5^+^ HF stem cells is sufficient to induce HF TAT.

To verify the role of Lgr5^+^ cells in WIH-A, we specifically depleted Lgr5^+^ cells in *Lgr5-Cre*:*R26*^*DTR/+*^ mice (P56–63) via the administration of TM and DT, and the absence of Lgr5^+^ HF stem cells almost completely blocked WIH-A on PWD-15 (Fig. 5d,e). In non-wounded skin, Lgr5^+^ cell depletion in the HFs resulted in a delay in depilation-induced HF TAT of ∼7 days (data not shown). The role of Lgr5^+^ cells in TNF-induced HF TAT was further validated by specifically knocking down *TNFR1* in Lgr5^+^ cells with shRNA, which markedly reduced TNF-induced p-AKT activity in cultured Lgr5^+^ cells derived from *Lgr5-EGFP* mice (Supplementary Fig. 6c). When intracutaneously injected, shRNA-TNFR1 was primarily detected in HF germ cells (Fig. 5d,e), the putative location of Lgr5^+^ stem cells, which verified the specificity of the shRNA, and led to the decreased expression of TNFR1 in Lgr5^+^ cells (Supplementary Fig. 5f). Consequently, shRNA-TNFR1 significantly reduced the number of WIH-A (Fig. 5f,g). To test the role of β-catenin signalling in WIH-A, which was upregulated in the wounds adjacent Lgr5^+^ HF stem cells *in vivo*, and demonstrated AKT signalling-dependent upregulation after TNF treatment *in vitro*, we developed *Lgr5-Cre:β-catenin*^*flox/flox*^ mice (P56–63). *β-catenin* knockout blocked WIH-A at PWD-15 (Fig. 5h,i), indicating that β-catenin signalling is indispensable for the contribution of Lgr5^+^ HF stem cells to WIH-A. Impressively, the number of neogenic HFs was dramatically decreased in Lgr5^+^ HF stem cells depleted mice (*Lgr5-Cre*:*R26*^*DTR/+*^ mice, wounding at P21–23) (Fig. 5j,k). To further evaluate the role of Lgr5^+^ cells in the WIHN, we developed *Lgr5-Cre:Rosa-mTmG* mice (TM treatment at P20 and P21, 1-cm diameter wound at P23) to perform Lgr5^+^ cell lineage tracing. First, lineage tracing indicated that all hair bulb cells of the anagen HFs (first anagen at P32, Anagen V) were Lgr5^+^ cell progeny (membrane tdTomato^−^/membrane GFP^+^, mT^−^/mG^+^) (Fig. 5l). These data further demonstrated the contribution of Lgr5^+^ cell progeny to all cycling structures of the HF at a single-cell resolution^4^. On the 7th day post-wounding, a substantial number of HF-derived Lgr5^+^ cell progeny mobilized to the wound area (Fig. 5m). Interestingly, we observed mT^−^/mG^+^ clones of the Lgr5^+^ progeny in the newly formed wound epidermis (Fig. 5n), suggesting that Lgr5^+^ cell progeny are involved in the basal layer and persistently contribute to the wound epithelium. Moreover, we found that the progeny of Lgr5^+^ cells (mT^−^/mG^+^) were detected in over 40% of neogenic HFs in the wound (Fig. 5o,p), and the number of Lgr5^+^ cell progeny in individual neogenic HFs differed greatly and ranged from <3% to over 90% of total HF cells (Fig. 5p). Collectively, based on our data, Lgr5^+^ cell progeny participate in and also make important contributions to HF neogenesis in wounds.

### AKT activation in Lgr5^+^ cells promotes hair regeneration

**Figure 6:**
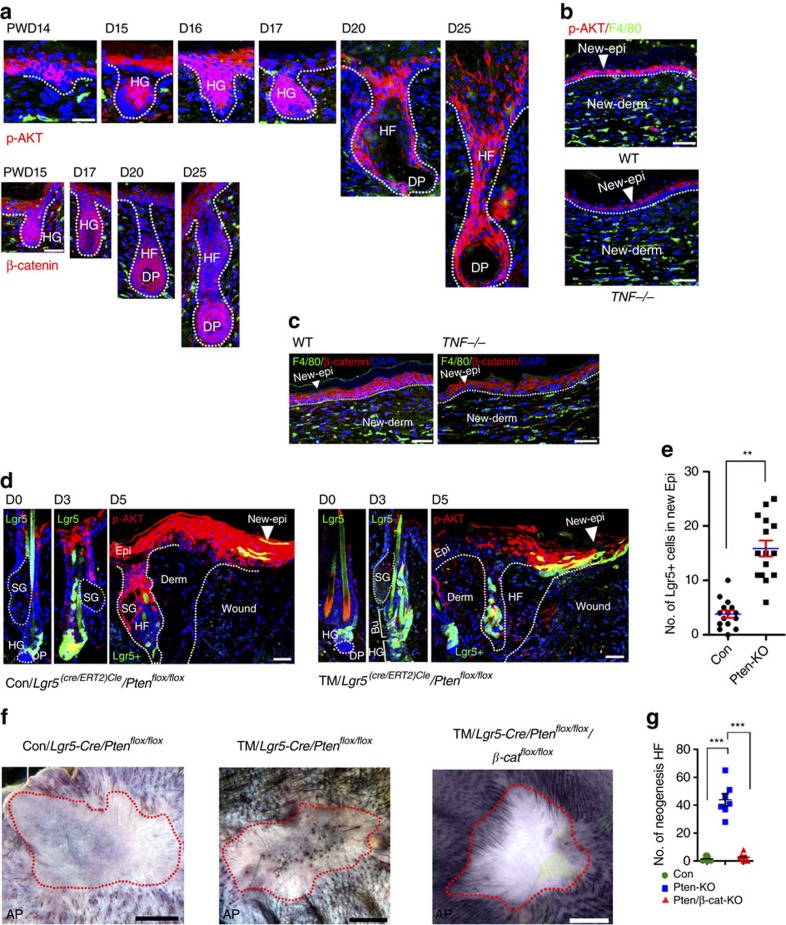
AKT activation in Lgr5^+^ cells promotes *de novo* hair regeneration. (**a**) New hair follicles in the wound began to form after PWD-14, and marked p-AKT was detected in the newly formed epidermis and in the hair germ (HG) that initiated hair follicle (HF) neogenesis. Similar to the p-AKT signal, abundant β-catenin was also detected. Scale bars, 30 μm. (**b**,**c**) p-AKT and β-catenin in the epidermis of re-epithelialized wounds of *TNFA*^*−*^*/*^*−*^ and WT mice at PWD-20. Scale bars, 50 μm. (**d**,**e**) Lgr5^+^ stem cells (GFP^+^) were present in the upper portion of the hair follicle at PWD-3 and in the re-epithelialized epidermis at PWD-5 in *Pten* knockout and the control mice (**d**). The number of GFP^+^ cells in the new epidermis of the two different groups was counted (**e**). *n*=15 tissue sections from 5 mice in each group were analysed. Scale bars, 50 μm. (**f**,**g**) *Pten* knockout in Lgr5^+^ cells promoted *de novo* HF regeneration, and synchronous knockout of *β-catenin* with *Pten* completely attenuated the *Pten* loss-induced hair follicle neogenesis in the wound (**f**). Then, the number of neogenic HFs in different groups was quantified (**g**). *n*=8 for tamoxifen-treated mice; *n*=9 for control mice. Scale bars, 1 mm. Bu, budge; SG, sebaceous gland; HG, hair germ; DP, dermal papilla. Data are expressed as the mean±s.e.m. **P<0.01. ***P<0.005, unpaired *t*-test, two-tailed.

To further define the role of AKT/β-catenin signalling in WIHN, we detected elevated levels of p-AKT and β-catenin in the cells of the re-epithelized epidermis and observed regenerating HFs (hair buds) in the wound (Fig. 6a). Consistently, p-AKT levels in the epidermis of the re-epithelized wound (PWD-20) in *TNFA*^*−/−*^ mice were much lower than those in wild-type mice (Fig. 6b). Similar changes were observed in β-catenin levels (Fig. 6c). The evaluation of Lgr5^+^ HF stem cells in TM-treated *Lgr5-Cre:Pten*^*flox/flox*^ mice after wounding revealed increased numbers of Lgr5^+^ (GFP^+^) cells in the upper portion of the HF at PWD-3 and in the re-epithelized epidermis at PWD-5 compared with the numbers seen in untreated mice (Fig. 6d,e). Importantly, TM-induced *Pten* loss in Lgr5^+^ HF stem cells in *Lgr5-Cre:Pten*^*flox/flox*^ mice (1-cm diameter wound at P35–42) promoted a number of neogenic HFs in the wound, while neogenic HFs were rarely observed in control mice (Fig. 6f,g). Impressively, the knockout of *β-catenin* expression with *Pten* in *Lgr5-Cre:Pten*^*flox/flox*^*:β-catenin*^*flox/flox*^ mice (P35–42) completely blocked *Pten* loss-induced HF neogenesis in the wound, suggesting that β-catenin is the crucial mediator in AKT-promoted HF neogenesis (Fig. 6f,g). According to a previous study, neogenic HFs in the wound are largely derived from stem cells in the epidermis^22^. Based on our data, enhanced AKT activation in Lgr5^+^ HF stem cells increases *de novo* HF regeneration in the wound. Our study to further support that HF stem cells play a crucial role in HF neogenesis in wounds.

## Discussion

WIH-A and WIHN were first described more than 60 years ago^6^,^20^,^37^,^38^, and both take place in a complicated micro-environment, particularly WIHN, which occurs simultaneously with inflammation, wound healing and HF neogenesis. Osaka *et al*. observed the crucial role of macrophages in WIH-A (ref. 11), Ralf Paus did pioneering work to investigate hair cycling and its related immune fluctuations^2^,^12^,^13^,^39^,^40^,^41^,^42^,^43^,^44^,^45^,^46^, and Cotsarelis and colleagues contributed landmark WIHN studies describing the important role of Wnt in WIHN (refs 22, 23). Perez-Moreno and colleagues found that skin-resident macrophages function as important mesenchymal regulators of HF stem cells function under physiological conditions, which identifies a novel link between macrophages and HF cycling^12^. Despite these findings, an understanding of the mechanism underlying these intriguing phenomena is lacking. In the current study, which is the first to explore of the role of macrophages in WIHN, the depletion of myeloid-macrophages (Ly6C^+^) completely diminished both WIH-A and WIHN. According to our data, TNF was essential for both WIH-A and WIHN; WIH-A increased in TNF-overexpressing mice, and TNF overexpression resulted in a substantial decrease in WIHN. To further explore the role of TNF in WIH-A and WIHN, we performed proteomic quantification of phosphorylation in TNF-treated Epi-SCs. AKT phosphorylation responded to TNF stimulation, and p-AKT levels were highly dependent on the treatment duration and the dose of TNF, which may partially explain why TNF overexpression resulted in decreased WIHN. Additionally, TNF treatment resulted in the AKT signalling-dependent accumulation of β-catenin and the phosphorylation of β-catenin-Ser552. *In vivo* analysis indicated that AKT signalling was highly activated in Lgr5^+^ HF stem cells after wounding, and Lgr5^+^ cell depletion blocked WIH-A and attenuated WIHN. Furthermore, *Pten* knockout in Lgr5^+^ cells promoted the transition from second telogen HFs to anagen HFs and dramatically promoted WIHN (5-week-old mice with 1-cm diameter wounds). Thus, the TNF-induced AKT/β-catenin signalling axis in Lgr5^+^ HF stem cells plays a dominant role in both WIHN and WIH-A. Collectively, this study achieved following new findings: (1) myeloid-derived macrophages, but not the tissue-resident macrophages are essential for the both WIH-A and WIHN. (2) Ly6C^+^ inflammatory macrophages showed distinctive gene expression pattern in cytokines compared with CX3CR1 resident macrophages and neutrophils. (3) TNF plays an important role in WIH-A and WIHN. (4) In epidermal stem cells TNF-α causes AKT phosphorylation and activation of β-catenin which appears independent of Wnt ligand binding. (5) TNF-induced p-AKT in Lgr-5^+^ HFSCs was crucial for WIH-A and WIHN.

The cellular basis for WIHN is not fully understood, and two alternative mechanisms, specifically a stem cell-based mechanism versus a reprogramming-based mechanism, have yet to be exhaustively tested^47^. Bugle HF stem cells (Krt15^+^) do not participate in HF neogenesis in wounds^22^, which argues against the stem cell-based mechanism. Although the HF stem cells located directly above the follicle bulge (Lgr6^+^) contribute 10% of neogenic HF within a wound^5^, Gli1^+^ cells become Epi-SCs during wound healing^48^, Lrig1^+^ cells dominantly contribute to *de novo* HF regeneration in reconstitution analysis^49^, and the HF stem cells beneath the follicle bugle (hair germ, Lgr5^+^) repopulate other stem cell compartments and extensively participate in wound re-epithelialization^4^,^50^,^51^,^52^, their contribution to WIHN has not been fully elucidated. In this study, Lgr5^+^ cell depletion resulted in >50% attenuation of WIHN numbers, and lineage tracing revealed Lgr5^+^ progeny in over 40% of neogenic HFs, suggesting that the Lgr5^+^ lineage cells may contribute to the initiation of neogenic HFs rather than just participating as cellular components. In support of this notion, *Pten* loss in Lgr5^+^ cells promotes extensive HF neogenesis in the wounds of 5-week-old mice, which otherwise are not capable of regenerating HF; thus, cell signalling activity, such as AKT/β-catenin signalling, in Lgr5^+^ cells is crucial for HF neogenesis. For the first time, we demonstrate the crucial role of HF stem cells in WIHN, and our data support the stem cell-based WIHN mechanism. Further elucidation of the earlier phase of *de novo* HF regeneration is likely to shed light on the role of HF stem cells in WIHN. HF bulge stem cells appear dispensable for the acute phase of wound re-epithelialization^53^, while at later phases of wound healing, many Lgr5^+^ cell progeny migrate to form the neo-epidermis^51^; however, the role of Lgr5^+^ cells in efficient wound repair has not yet been addressed. A more accurate wound re-epithelialization model is desired to quantitatively analyse the contribution of Lgr5^+^ cells in wound re-epithelialization.

According to previous studies, both epidermal and dermal Wnt activation are necessary for HF placode formation^54^,^55^. FGF9 from γδT cells in skin wounds induces dermal Wnt activation^23^, and exposure to DKK1, a Wnt antagonist, completely blocks WIHN^22^. However, in this study, DKK1 did not obviously inhibit TNF-induced β-catenin signalling activity in Epi-SCs. In addition, TNF induced AKT-dependent phosphorylation of β-catenin-Ser552; thus, the increased β-catenin signalling in the Epi-SCs appears to depend on the TNF/p-AKT/p-β-catenin-Ser552 signalling axis rather than increased Wnt expression, which is thought to activate β-catenin by promoting p-GSK-3β(Ser9) phosphorylation and a decline in the phosphorylation of β-catenin (Ser33/37/Thr45)^56^,^57^. However, Wnt ligands may play an essential role in epidermal and dermal interactions during the earlier phase of HF neogenesis, similar to HF morphogenesis during the embryonic stage^25^,^26^,^27^,^58^. Although our study does not address how TNF/TNFR1 actually activates AKT signalling (p-AKT), it will be interesting to obtain further insight into how TNF induces p-AKT signalling in a dose-dependent manner and how negative feedback is regulated.

During inflammation, all macrophages are activated and differentiate into M1-like inflammatory cells following interactions with pathogenic and damaged signals/insults in the surrounding microenvironment^18^,^19^,^59^. Therefore, both Ly6C^+^ myeloid-derived macrophages and tissue-resident CX3CR1^+^ macrophages that infiltrate the wound site are likely activated as M1 macrophages, and in this study, we analysed the role of these two sub-populations of M1 macrophages in both WIH-A and WIHN. Because macrophages recruited from the blood during the post-inflammatory phase lose Ly6C expression and become Ly6C^−^ cells, subsequently differentiating into M2 macrophages, the M1 to M2 transition may inevitably take place during wound healing^60^,^61^. As WIH-A occurs during an early phase of wound healing, Ly6C^+^ macrophages clearly make major contributions to this process. In contrast to WIH-A, WIHN takes place during the later phase of wound healing (PWD 14–21)^23^. According to our data, the depletion of Ly6C^+^ macrophages blocked WIHN, but it is unlikely that Ly6C^+^ macrophages exert a direct influence on WIHN, while Ly6C^+^ macrophages may exert an indirect influence on WIHN by transforming into Ly6C^−^ and TNF-expressing macrophages. This notion is consistent with the co-localization of macrophages with TNF at PWD-14 in this study. More work must be performed to evaluate the concrete role of Ly6C^−^/F4/80^+^ macrophages in WIHN, and the relationships among Ly6C^−^ macrophages, M2 macrophages and CX3CR1^+^ resident macrophages during the later stage of wound healing. Specifically, the evaluation of Ly6C^+^ macrophage lineage mice during wound healing will help us to understand the efficiency of these myeloid-macrophage activated M1 macrophages during the earlier stage of wound healing as well as the efficiency of their transition to M2 macrophages during the later stage of wound healing. Furthermore, the specific depletion of M2 macrophages during the later stage of wound healing will allow us to understand the role of M2 macrophages in WIHN.

HF stem cells are composed of different stem cell subpopulations, among them the Lgr5^+^ cells are located in secondary hair germ, Krt15^+^ cells (most co-express CD34^+^) are located in the bulge, and Lgr6^+^ cells are above the bulge and show similar capacity in regenerating the hair follicle and epidermis^3^,^4^,^5^. Viljar and colleagues found that CD34^+^ cells were less capable in regenerating the HF, suggesting that Lgr5^+^ cells are in the upper level of the HF stem cell hierarchy (higher degree of stemness) than CD34^+^ cells. However, the relationship among Lgr5^+^, CD34^+^ and Lgr6^+^ HFSC is not fully understood.

In summary, our study illuminates the role of macrophage-derived TNF in the wound as an inducer of both WIH-A and WIHN, and TNF activates HF stem cells via the TNF/p-AKT/p-β-catenin-Ser552 signalling axis. By highlighting the crucial role of AKT signalling in HF stem cell activation, we also observed the ability of elevated AKT signalling (*Pten* loss) in Lgr5^+^ cells to promote WIHN, which further supports the dominant role of HF stem cells in WIHN.

## Methods

### Mice

C57/B6 mice (6-week-old, female) were purchased from Guangdong Medical Laboratory Animal Center, Guangzhou, China. *Lgr5-GFP-Cre-ERT2* (*Lgr5-Cre*) mice were obtained from Jackson Laboratory (stock no.: 008875). The mice were carefully analysed and confirmed to have no aberrant phenotypes before being crossed to *Pten*^*flox/flox*^ mice (a gift from Dr Hong Wu at the University of California, Los Angeles) to obtain *Lgr5-Cre:Pten*^*flox/flox*^ mice. To knock out *Pten* in Lgr-5 cells, *Lgr5-Cre*: *Pten*^*flox/flox*^ mice aged 8–9 weeks received an intraperitoneal injection of 200 μl of TM (Sigma) in corn oil at a dose of 10 mg ml^−1^; to knock out *Pten* regionally in the skin, 20 μl of TM at a dose of 10 mg ml^−1^ was intracutaneously injected. *Lgr5-Cre:Pten*^*flox/flox*^ mice were crossed with *β-catenin*^*flox/flox*^ (B6.129-Ctnnb1^tm2Kem^/KnwJ, provided by Dr Zhenge Luo, Institute of Neuroscience, CAS) mice to obtain *Lgr5-Cre:Pten*^*flox/flox*^*:β-catenin*^*flox/flox*^ mice. *Lgr5-Cre* mice were crossed with *β-catenin*^*flox/flox*^, *R26*^*iDTR*^ (Provided by Biocytogen, Beijing, China) and *Rosa-mTmG* mice (JAX mice, Stock no.: 007576) to obtain *Lgr5-Cre:β-catenin*^*flox/flox*^, *Lgr5-Cre:R26*^*iDTR/+*^ and *Lgr5-Cre:Rosa-mTmG* mice. *Cx3cr1*^*CreER*^*:R26*^*iDTR/+*^ mice were provided as a gift from Dr Wenbiao Gan at New York University. To induce the depletion of CX3CR1^hi^ cells, *Cx3cr1*^*CreER/+*^*: R26*^*iDTR/+*^ mice received sequential treatments with TM and DT (Sigma). A total of 10 mg of TM was given in corn oil to mice aged 7–8 weeks by oral gavage every 24 h for 3 days, followed by the intraperitoneal injection of 1 μg of DT in phosphate-buffered saline (PBS) every 24 h for another 3 days^62^. *LysM-Cre* mice (provided as a gift from Dr Yuqing Huo at Peking University Shenzhen Graduate School) were crossed with *R26*^*iDTR*^ mice to obtain *LysM-Cre:R26*^*iDTR/+*^ mice. IL-6 knockout mice (IL6-KO, B6.129S2-Il6^tm1Kopf/^J), TNF-α knockout mice (TNFα-KO, B6.129S6-Tnf^tm1Gkl/^J), *TNFR1*^*−/−*^ mice (Stock Number: 002818) and *TNFR2*^*−/−*^ mice (Stock Number: 002620) were obtained from Jackson Laboratory. *LysM*^*cre/+*^*:TNF-a*^*flox/flox*^ (MN-TNFα-KO) mice were generated in Dr Sergei Nedospasov's laboratory at the Engelhardt Institute of Molecular Biology, Russian Academy of Sciences, Russia). Transgenic (Tg)-TNF mice (that overexpressed *TNFA*) were created by the Guangdong Laboratory Animals Monitoring Institute. *Tnf-Luc-eGFP* mice, whose luciferase expression was driven by the *TNFA* promoter, were obtained from Shanghai Biomodel Organism Science & Technology Development. Bioluminescent imaging of luciferase expression in the mice was performed using an IVIS 100 Imaging System (Caliper-Perkin Elmer, Hopkinton, MA, USA) after the animals received an intraperitoneal injection of 200 μl of D-luciferin at a concentration of 15 mg ml^−1^ in Dulbecco's PBS. Since the wound healing, immune status and key immune responses of murine skin are strictly hair cycle dependent^46^,^63^,^64^, and for the proper control of these potential differences, we used 7–9 weeks old mice (in second telogen hair phase) in all experiments for studies of WIH-A. To ensure all the mice used for WIH-A are in second telogen hair phase, mice were checked for the skin colour and skin thickness before wound (based on the hair cycle stages criteria established by Ralf Paus^65^). In addition, we used littermate mice for control in all genetic mice model involved experiment. For the mice use in WIHN, we also strictly same both date or littermate mice. Mice were randomly divided into groups using a random-number table. The animals were maintained in a temperature-controlled environment (20±1 °C) with free access to food and water. All procedures were performed with the approval of the Animal Ethics Committee of Tsinghua University.

### Excisional wounds in mice

C57BL/6 (8 weeks, female) mice were anesthetized with an intraperitoneal injection of sodium pentobarbital (50 mg kg^−1^). One or two symmetrical full-thickness skin wounds 2–10 mm in diameter were created on the back (without hair removal) with a skin biopsy punch^66^. Firstly, put the mouse on its side on a sterile sheet. Pull the dorsal skin of the chest from the midline with fingers, and punch through the folded skin (both layers) with a 5-mm diameter sterile biopsy punch to create two symmetrical full-thickness excisional wounds besides the midline. To examine the effects of PI3K and AKT inhibition on HF TAT, the dorsal hairs were shaved and depilated with a depilatory cream, and inhibitors were applied to the skin at the depilated area.

### Inhibitors and cytokines

A CCR2 antagonist (CAS 445479-97-0, Santa Cruz Biotechnology) was dissolved in dimethyl sulfoxide (DMSO) and injected intraperitoneally at 10 mg kg^−1^ body weight every 3 days for 9 days. Perifosine (KRX-0401), a synthetic substituted heterocyclic alkylphospholipid, was provided by Selleck Chemicals, USA. A total of 30 mg of perifosine incorporated in a 30-g lipid emulsion or an equal amount of lipid emulsion alone (control) was smeared onto the dorsal skin of 7–8-week-old mice after hair depilation at a dose of 1 g per mouse per day for 10 consecutive days. Twenty days after hair removal, HF TAT was analysed. A total of 50 μl of LY294002 (PI3K inhibitor, BioVision, USA) in Matrigel (growth factor reduced, BD Biosciences) at a concentration of 200 μM, or an equal amount of Matrigel alone (control), was injected intracutaneously into the backs of 7–8-week-old mice after hair depilation every 48 h for 15 days (7 injections in total). Twenty days after hair removal, HF TAT was analysed. bpV (phen), a PTEN inhibitor (BioVision, USA), was incorporated into nanoparticles (NPs) to prolong release^67^. In brief, 10 mg PLGA/TPGS mixture was dissolved in 5 ml acetone and injected into aqueous solution under stirring to form homogenous emulsion. After solvent evaporation, 1 ml of resulting NPs was taken for size and zeta potential determination, and the rest was centrifuged and washed to obtain NP pellets. The NPs were resuspended and frozen-dry for 48 h to generate NP powders. For preparation of bpV/TPGS-loaded PLGA NPs, 0.2 mg bpV was dissolved along with PLGA/TPGS mixture and the fabrication process was exactly as described above. The bpV-loaded NPs was suspended in 50 μl of PBS or an equal volume of NP-PBS (control) was injected intracutaneously into the backs of 7–8-week-old C57BL/6 mice. Five milligrams of lenalidomide (Selleck Chemicals, USA), a TNF- secretion inhibitor, in DMSO (Sigma) at a concentration of 50 mg ml^−1^, or QNZ (Selleck Chemicals, USA), a TNF-α production inhibitor, which was dissolved in 0.5% hydroxyethyl cellulose, was injected intraperitoneally into 7–8-week-old C57BL/6 mice every day for 10 days; control mice received injections of an equal volume of DMSO or hydroxyethyl cellulose accordingly. Dexamethasone (Sigma) at a dose of 10 mg kg^−1^ body weight (>50 mg kg^−1^ dexamethasone or excessive dexamethasone was topically applied via smear, the agent showed hair cycle-modulatory effects. However, low dose of dexamethasone (10 mg kg^−1^, i.p. injection) did not show any hair cycle-modulatory effects, but could efficiently inhibit inflammation reaction after wound) or PBS (control) was injected intraperitoneally daily for 10 days. JSH-20 (Selleck Chemicals, USA), an NF-κB inhibitor, was dissolved in 0.5% hydroxyethyl cellulose and intraperitoneally injected at a dose of 0.5 mg daily for 10 days; an equal volume of 0.5% hydroxyethyl cellulose was injected into control mice. Recombinant Mouse TNF-α (carrier-free) (575204, BioLegend).

### Immunostaining

Frozen skin tissue sections were incubated with different primary antibodies at 4 °C overnight; antibodies included anti-CD49f-biotin (GoH3, 1:150, BioLegend), anti-Ki67 (20Raj1, 1:100, eBioscience), anti-Akt (GTX28932, phospho Ser473, 1:150, GeneTex), anti-Akt (Akt 1+2+3) (GTX121937, 1:150, GeneTex), anti-CD34-biotin (RAM34, 1:150, eBioscience), anti-Lgr-5 (ab137484, 1:200, Abcam), anti-CD45 (30-F11, 1:150, BioLegend), anti-CD45-Biotin (30-F11, 1:150, BioLegend), anti-F4/80-Biotin (BM8, 1:150, BioLegend), anti-F4/80 (BM8, 1:150, BioLegend), anti-Ly6C-Biotin (RB6-8C5, 1:200, BioLegend), anti-Ly6C (HK1.4, 1:200, BioLegend), anti-MHC II (14-4-4S, 1:200, eBioscience), anti-TNF-α (1F3F3D4, 1:100, eBioscience), anti-TNFR1-Biotin (55R-170, 1:100, BioLegend), anti-β-catenin (C2206, 1:150, Sigma) and anti-Phospho-β-Catenin (Ser552) (5651S, 1:100, CST Signaling). Secondary antibodies with different fluorescence conjugates (FITC, Cy3/TRITC and Alex Fluor 647) were used for detection, and cells were visualized with an Olympus FV1000 confocal microscope.

### Isolation of epidermal stem cells and inflammatory cells

Mouse dorsal skin was harvested. The tissue was cut into 2–3 mm^2^ pieces, washed three times in Hank's balanced salt solution (HBSS), and digested with 0.3% dispase II (Sigma) for 90 min at 37 °C. The epidermis was manually removed from the tissue. Epithelial stem cells were isolated based on their highly adhesive properties. Briefly, the epithelial layer was cut into a slurry and treated with 0.2% collagenase I (Sigma) for 60 min at 37 °C with shaking and was filtered through a 40-μm cell strainer. The cells were seeded in tissue culture dishes coated with 50 mg ml^−1^ collagen I (Sigma) and were incubated in CnT-07 PCT Epidermal Keratinocyte Medium (CnT-07; CELLnTEC Advanced Cell Systems, Switzerland) containing supplements provided by the manufacturer for 60 min. The non-adherent cells were removed, and adherent cells were maintained.

To isolate cells from wounds, mouse wound single-cell suspensions were prepared. Briefly, the wound, along with a small amount of surrounding skin tissue, was excised with a biopsy punch and digested with 0.3% dispase II (Sigma) for 90 min at 37 °C. The epidermis was manually removed from the tissue. The dermis was minced and incubated in a digestion buffer that contained hyaluronidase (1 mg ml^−1^), collagenase D (1 mg ml^−1^), and DNase (150 units per ml) in a 37 °C shaking water bath for 2 h. The digest was filtered through a 70-μm nylon cell strainer, pelleted and resuspended in PBS that contained 1% bovine serum albumin (BSA, Sigma). Macrophages and neutrophils were isolated using a biotinylated anti-F4/80 antibody and a biotinylated anti-Ly6G (Gr-1) antibody (BioLegend), respectively, and streptavidin microbeads (Miltenyi). Cells that were negative for F4/80 and Ly6G were collected and designated ‘other cells'.

Mouse blood neutrophils were isolated with Percoll. Add 5.0 ml Percoll centrifuge tube, and carefully layer 5.0 ml of blood over the separation media. Centrifuge at 500 RCF for 35 min at room temperature. Carefully remove the top layers using a pipette Dispose of these layers. Carefully pipette the layer of neutrophils and all of the isolation media beneath the neutrophils. Place the solution into a clean centrifuge tube. Dilute the neutrophil solution to 10 ml with HBSS without Ca^2+^/Mg^2+^. Centrifuge the neutrophil solution at 350 RCF for 10 min. Aspirate the supernatant and discard. Resuspend the pellet in 250 μl HBSS/BSA Solution (2% BSA). The blood was collected from C57/B6 mice via cardiac puncture after anesthesia. Murine bone marrow-derived macrophages were isolated from the femurs of C57/B6 or TNF-KO mice. Briefly, cut off the hind legs at the hip joint with scissors, leaving the femur intact. Remove excess muscle from legs by holding end of bone with forceps and using scissors to push muscle downward away from forceps. Using sharp scissors or razor blade soaked in ethanol, carefully sever leg bones proximal to each joint. Attach 10-ml syringe to 25-G needle and fill with cold sterile wash medium. Insert needle into bone marrow cavity of femur. Flush bone cavity with 2–5 ml of the wash medium, until bone cavity appears white. Allow wash medium to collect in a sterile 50 ml conical centrifuge tube on ice. Centrifuge cells 10 min at 500*g*, room temperature. Discard supernatant. Resuspend cell pellet in DMEM/F12-10 medium supplemented with 100 U ml^−1^ recombinant macrophage colony-stimulating factor (ProSpec) for 7 days and assessed by flow cytometry to analyse the expression of the macrophage markers F4/80 and CD11b. To activate macrophages, the cells were treated with 20 ng ml^−1^ lipopolysaccharide (Sigma) for 24 h.

### Flow cytometry

Cells were suspended in PBS containing 1% BSA (Sigma) at 10^6^ cells per milliliter. Nonspecific binding to Fc receptors was blocked by pre-incubation with anti-CD16/32 (BioLegend). Cell aliquots (100 μl) were incubated with CD11b-FITC (clone M1/70), CD45-AlexFluor647 A700 (clone 30-F11), F4/80-PE, Ly6C- AlexFluor647, MHC II-FTIC or control isotype IgG on ice for 30 min. All antibodies were purchased from BioLegend. Ten thousand events were analysed by flow cytometry (BD Biosciences) using CellQuest software.

### Microarray analysis

Ly6C^+^/F4/80^+^ inflammatory macrophages, CX3CR1^+^/F4/80^+^ tissue-resident macrophages and Ly6G^+^/F4/80^−^ neutrophils were isolated from 3-day wounds in C57/B6 mice aged 8 weeks via cell sorting. Total RNA was extracted from the cells with TRIzol (Invitrogen). A total of 10 μg of labelled cRNA was hybridized at 45 °C for 16 h to the mouse genome array MOE430A2.0 (Affymetrix). Processed chips were read with an Agilent G2565CA Microarray Scanner. Scanned microarray images were imported into GeneSpring GX Software to generate signal values and absent/present calls for each probe-set using the MAS 5.0 statistical expression algorithm to identify genes that were differentially expressed.

### Western blotting

Cell lysates were prepared in a lysis buffer containing 1% Triton X-100, 1% deoxycholic acid, 2 mM CaCl_2_ and protease inhibitors (10 μg ml^−1^ leupeptin, 10 μg ml^−1^ aprotinin, 1.8 mg ml^−1^ iodoacetamide and 1 mmol l^−1^ phenylmethyl sulfonyl fluoride) and were quantified with a BCA protein assay kit (Pierce). Equal amounts of total protein were subjected to electrophoresis on 12% Bis-Tris gels, transblotted onto nitrocellulose membranes and probed with different primary antibodies: anti-AKT (phospho Ser473, 1:1,500, GeneTex, GTX28932), anti-pan-AKT [N3C2] (1:1,500, GeneTex, GTX121937), anti-TNFR1 (BioLegend, 113403, 1:1,000), anti-phospho-β-catenin (Ser552) (CST Signaling, 5651S, 1:1,000), anti-phospho-β-catenin (Ser33/37/Thr41) (CST Signaling, 9561S, 1:1,000), anti-phospho-GSK-3β (Ser9) (CST Signaling, 5558S, 1:1,000), and anti-phospho-β-catenin (Ser675) (CST Signaling, 4176S, 1:1,000). A peroxidase-conjugated secondary antibody (GeneTex) was then applied. Immunoreactive bands were detected using an ECL kit according to the manufacturer's instructions. Subsequent reprobing with an anti-glyceraldehyde-3-phosphate dehydrogenase (GAPDH) antibody was performed as an internal loading control.

### TCF/LEF-dependent luciferase reporter assay

Epi-SCs were isolated and plated in 12-well culture plates. When the cells reached 70% confluence, they were transfected with an inducible dual luciferase reporter plasmid (QIGEN, CCS-018L) containing TCF/LEF DNA binding sites, a negative control (a non-inducible luciferase construct to eliminate background) or a positive control vector (a GFP-expressing construct used for visual confirmation and transfection optimization) using Oligofectamine according to the manufacturer's protocol (Invitrogen, Lipofectamine LRX & PLUS Reagent). Twenty-four hours after transfection, cells were serum-starved for 8 h and then treated with TNF-α and inhibitors for another 8 h. To determine β-catenin activity, cells were lysed in a luciferase lysis buffer (Promega). The lysates were aliquoted and analysed for firefly luciferase and Renilla activities using a luminometer. All experiments were performed in triplicate.

### Real-time PCR

Total RNA was extracted with TRIzol (Invitrogen) following the manufacturer's instructions. First-strand cDNA was prepared via reverse transcription with Superscript II reverse transcriptase (Invitrogen) and oligo (dT) primers and was stored at 20 °C. Real-time polymerase chain reaction (Real-time PCR) was performed using SYBR Premix Ex Taq II on an ABI 7300 QPCR System. As an internal control, GAPDH levels were quantified in parallel with target genes. Normalization and fold changes were calculated using the ΔΔCt method. Primers that used to amplify murine genes are provided in Supplementary Table 2.

### RNAi

Lentiviral vectors contained LGR5-mTNFR1 shRNA. The shRNA sequence targeting murine TNFR1 was AGATCTCTCCTTGCCAAGCTTCAAGAGAGC-TTGGCAAGGAGAGATCT. The sequence of the murine *Lgr5* promoter was obtained from Genecopoeia (USA). The *Lgr5* promoter and mTNFR1 shRNA sequences were synthesized by GenScript (USA). The Lgr5-mTNFR1-shRNA-polyA sequence was initially cloned into a pUC57 vector and was then transferred into a pLVX-shRNA2-m lentiviral vector, resulting in a pLVX-Lgr5-mTNFR1-shRNA-CMV-ZsGreen plasmid. The plasmid was transfected into 293 cells for viral production. pLVX-Lgr5-CMV-ZsGreen plasmid viruses were produced for use as mock controls. Lgr-5^+^ HF stem cells isolated from *Lgr5-Egfp* mice were seeded into 6-well plates and were infected the next day with 10 μl of TNAR1 shRNA or mock lentiviruses and 8 μg ml^−1^ polybrene. Five days later, TNFR1 expression was assessed via western blotting and immunofluorescence to determine shRNA efficiency. To knock down *Tnfr1 in vivo*, 100 μl of TNFR1 shRNA or mock lentiviruses was mixed with an equal volume of Matrigel and intracutaneously injected into the skin on the back of 8-week-old mice every 48 h for a total of 3 injections. The day following the last viral injection, wounds were created at the injection sites with a 5-mm biopsy punch to assess injury-induced HF stem cell activation and follicle TAT.

### NP-encapsulated siCCR2

An siRNA duplex with the sequences 5′-uGcuAAAcGucucuGcAAAdTsdT-3′ (sense) and 5′-UUUGcAGAGACGUUuAGcAd-TsdT-3′ (anti-sense), which we previously shown to exhibit superior efficiency among numerous candidates, was employed to silence murine *CCR2* (ref. 68). For *in vivo* experiments, the duplex was formulated into lipid NPs as follow^68^. Nanoparticles were prepared with the cationic lipid C12–200, disteroylphosphatidyl choline, cholesterol and PEG-DMG using a spontaneous vesicle formation procedure. Lipids were dissolved in 90% ethanol solution and mixed with siRNA solution (25 mM citrate, pH 3 ratio) at fixed speed (1:1 ratio) and diluted immediately with PBS to final 25% ethanol. The ethanol was then removed and the external buffer replaced with PBS (155 mM NaCl, 3 mM Na_2_HPO_4_, 1 mM KH_2_PO_4_, pH 7.5) by dialysis. Particle size and zeta potential were determined using a Malvern Zetasizer NanoZS. siRNA content was determined by ion exchange high-performance liquid chromatography (Agilent) assay using DNAPac Pa200 column.

### Proteomic analysis of phosphorylation

Proteomic quantification of phosphorylation was performed as previously reported^69^,^70^. Briefly, cell lysates were prepared from cultured murine epidermal stem cells with and without TNF-α treatment, were reduced with 10 mM DTT for 1 h at 37 °C and were alkylated with 20 mM IAA for 45 min at room temperature in the dark. After trypsin digestion, peptides were desalted with a Strata X C18 SPE column (Phenomenex), vacuum-dried, and reconstituted in 0.5 M TEAB (6-plex TMT kit) according to the manufacturer's instructions. Samples were then fractionated by high-pH reverse-phase HPLC using an Agilent 300Extend C18 column (5-μm particles, 4.6-mm ID, 250-mm length). Peptide mixtures were incubated with an IMAC microsphere suspension with vibration and then eluted. The supernatant, which contained phosphopeptides, was collected and lyophilized for LC–MS/MS analysis (Acclaim PepMap 100, Thermo Scientific). Peptide separation was performed using a reversed-phase analytical column (Acclaim PepMap RSLC, Thermo Scientific) with a linear gradient of 4–22% solvent B (0.1% FA in 98% ACN) for 50 min, 22–35% solvent B for 12 min, 35–80% solvent B for 4 min and then holding at 80% for the last 4 min at a constant flow rate of 280 nl min^−1^ on an EASY-nLC 1000 UPLC system. The resulting peptides were analysed with a Q ExactiveTM Plus hybrid quadrupole-Orbitrap mass spectrometer (Thermo Fisher Scientific). The MS data were validated for bioinformatics analysis.

### Statistical analyses

Results are expressed as mean±s.e.m. unless stated otherwise. Statistical comparisons between two groups were evaluated by Student's *t*-test, unpaired *t*-test, two-tailed. A probability (*P*) value of<0.05 was considered to indicate statistical significance.

### Data availability

Microarray data that support the findings of this study have been deposited in Gene Expression Omnibus with the primary accession code GSE74783. The authors declare that all other data supporting the findings of this study are available within the article and its Supplementary Information Files or from the corresponding author upon request.

## Additional information

**How to cite this article:** Wang, X. *et al*. Macrophages induce AKT/β-catenin-dependent Lgr5^+^ stem cell activation and hair follicle regeneration through TNF. *Nat. Commun.* **8,** 14091 doi: 10.1038/ncomms14091 (2017).

**Publisher's note:** Springer Nature remains neutral with regard to jurisdictional claims in published maps and institutional affiliations.

## Supplementary Material

Supplementary Information Supplementary Figures and Supplementary Tables
