# Supplementary material for: Macrophages induce AKT/β-catenin-dependent Lgr5+ stem cell activation and hair follicle regeneration through TNF
Source: Nat Commun. 2017 Mar 27;8:14091. doi: 10.1038/ncomms14091 (PMC5378973; doi:10.1038/ncomms14091)
Supplement: Supplementary Information — Supplementary Figures and Supplementary Tables [file ncomms14091-s1.pdf]

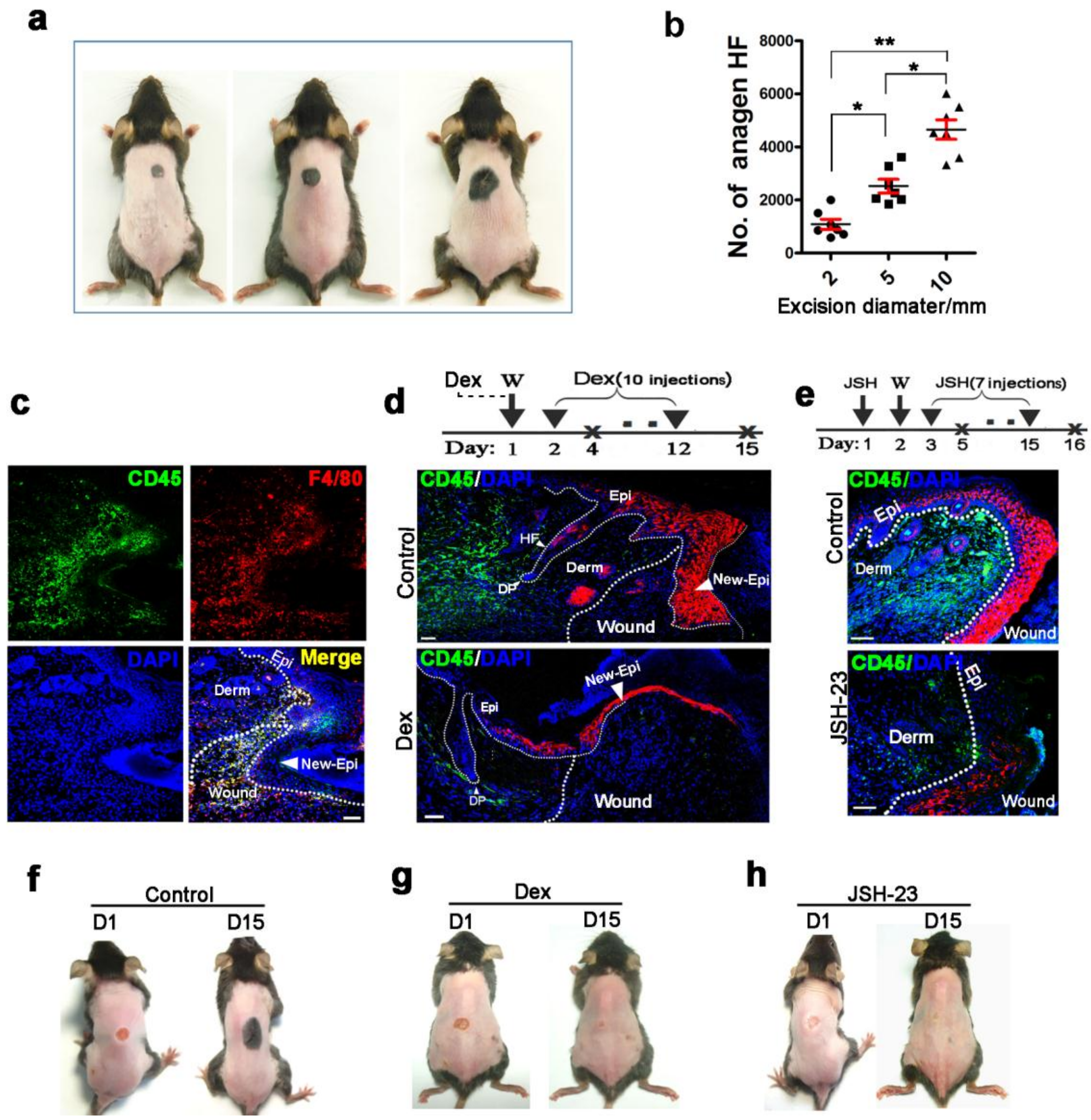

Supplementary Figure 1.

(a, b) The number of anagen follicles was proportional to the size of excisional wounds. Representative images showing areas of anagen follicles around the wound at the 15th day post-wounding (PWD-15) (a). The numbers of anagen follicles in different groups were quantified (b). n = 7 for each wound size. Data are expressed as the mean ± s.e.m. *P<0.05, **P<0.01, unpaired t-test, two-tailed.

(c) Immunofluorescence (IF) analysis showed densely populated CD45$^+$ leukocytes in the wounded skin. Double staining for CD45 and F4/80 showed that macrophages accounted for the majority of the inflammatory cells in the wound-adjacent tissue at PWD-3. For all IF analyses, representative images from 8-16 tissue sections of wounds in 4-6 mice are shown.

(d, e) IF analysis showed that treatment of mice with dexamethasone (Dex) (d) or JSH-23 (e) substantially inhibited the infiltration of CD45$^+$ leukocytes in the wound tissue (PWD-3). Epi, epidermis; Derm, dermis. Scale bars, 50 μm.

(f-h) Dex or JSH-23 treatment completely abolished the wounding-induced telogen-anagen HF transition as assessed at PWD-15. n = 8 mice for each group.

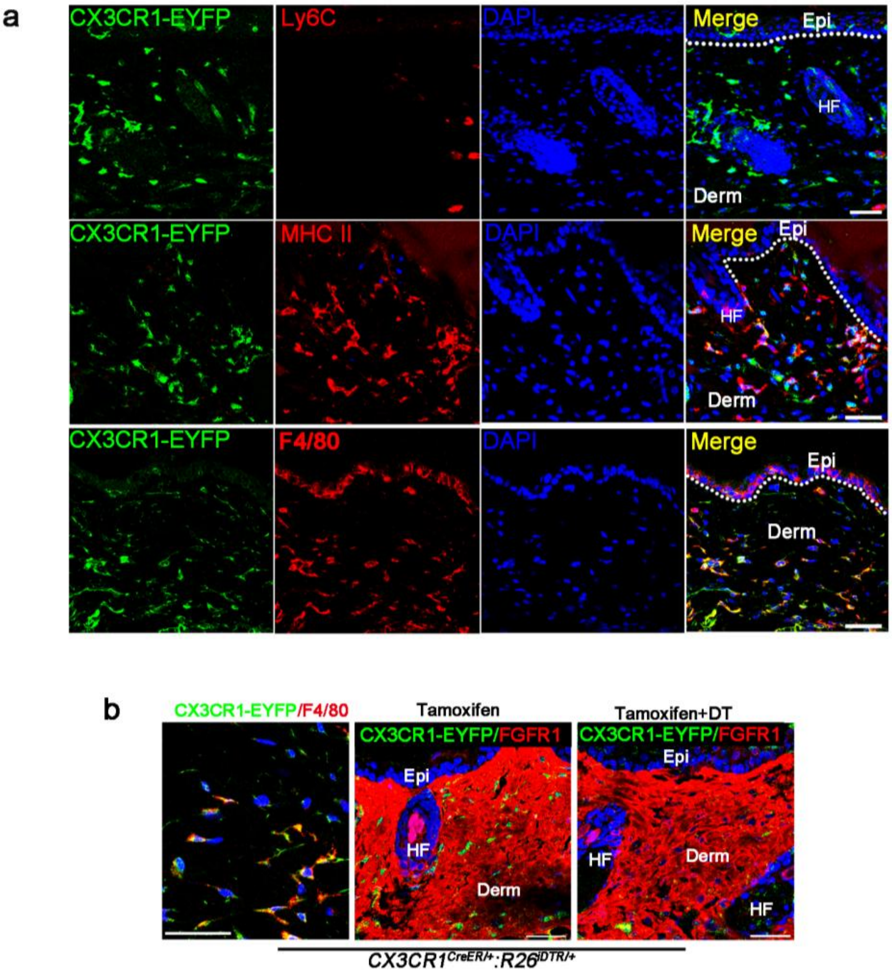

Supplementary Figure 2.

(a) IF analysis of normal skin of *CX3CR1*$^{CreER/+}$*:R26*$^{iDTR/+}$ mice showed that in the unwounded skin, the CX3CR1^{+} residential macrophages are widely distributed in the skin tissue, while the Ly6C^{+} myeloid-macrophages are rarely detected (upper channel). CX3CR1-YFP cells are positive for F4/80 and largely co-localized with MHC II^{+} cells (lower channel).

(b) After treatment of the mice with tamoxifen (TM) and diphtheria toxin (DT), CX3CR1-YFP^{+} macrophages (F4/80^{+}) in the skin were barely detected. Five *CX3CR1*$^{CreER/+}$*:R26*$^{iDTR/+}$ mice were analyzed before and after TM and DT treatment. Scale bars, 50 μm.

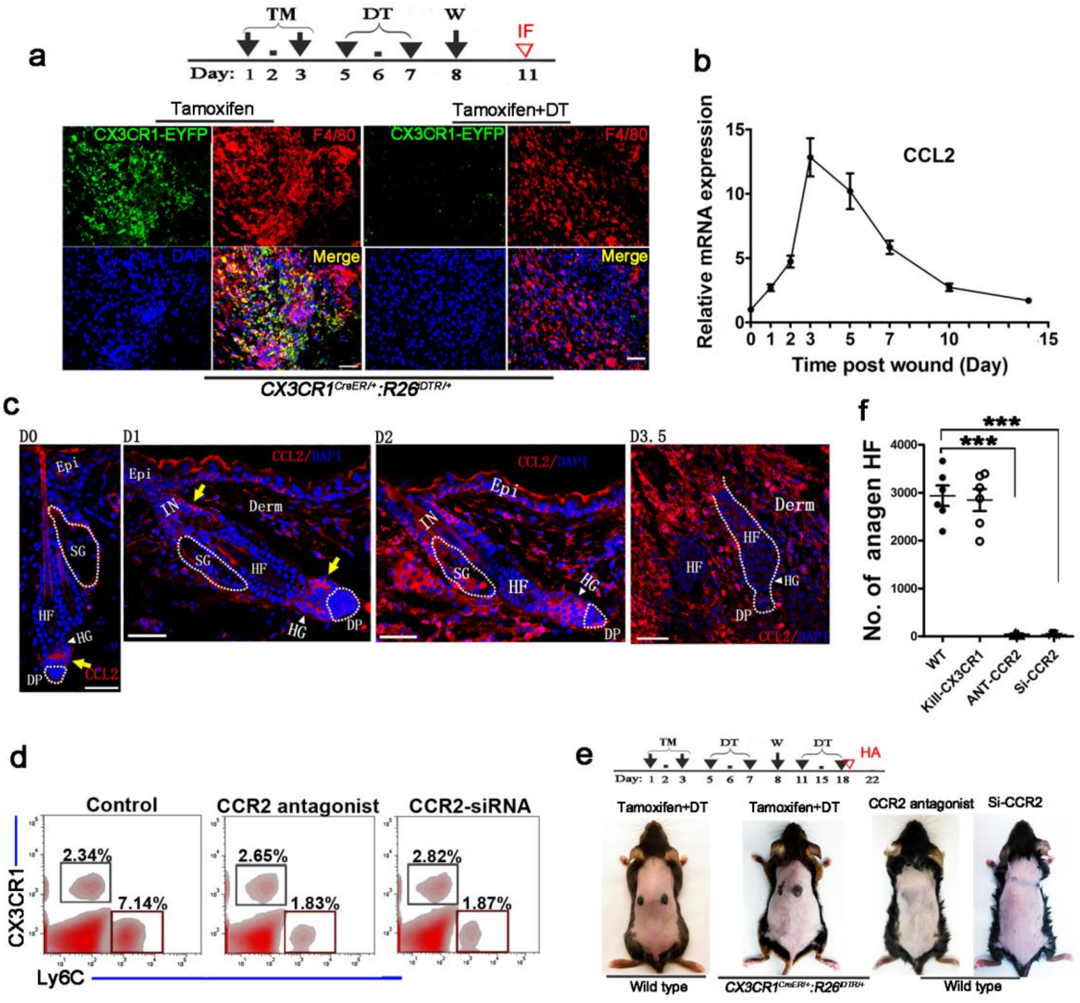

Supplementary Figure 3.

(a) IF analysis showed that CX3CR1-YFP macrophages decreased to barely detectable levels in the wounds (PWD-3) of *CX3CR1*$^{CreER/+}$*:R26*$^{iDTR/+}$ mice after TM and DT treatments. Scale bars, 50 μm.

(b) Real-time PCR from wound adjacent tissues (2 mm in width) revealed the kinetics of *CCL2* expression at different times post-wounding (PWD: 0, 1, 2, 3, 5, 7, 10, 14). (Gene expression was normalized to GAPDH with 40 cycles, data are represented as the mean ± SD, and n = 3.)

(c) IF analysis of the wound adjacent tissue at different times for the expression of CCL2. In unwounded skin, CCL2 was detected in the hair germ (HG) (yellow arrow); at PWD-1, CCL2 was detected in the HG, hair follicle infundibulum (IN) and some epidermal cells and dermal cells. At PWD-3.5, the levels of CCL2 increased in dermal cells but decreased in the HG and IN. Scale bars, 50 μm.

(d) Flow cytometry analysis indicated that the treatment of mice with CCR2 antagonist or CCR2-siRNA decreased Ly6C^{+} macrophages in the wound from 7.14±0.4% to 1.83±0.3% and 1.87±0.4%, respectively.

(e, f) Treatment of *CX3CR1*$^{\mathrm{CreER/+}}$*:R26*$^{\mathrm{iDTR/+}}$ mice with TM and DT showed little influence on WIH-A, while the inhibition of Ly6C^{+} macrophage infiltration with CCR2 antagonist or Si-CCR2 attenuated wounding-induced HF TAT (e). The number of anagen hair follicles in each group was counted (f). Data are expressed as the mean ± s.e.m. ****P*<0.005, unpaired t-test, two-tailed. Wild-type mice (C57BL/6 mice), *n* = 8; *CX3CR1*$^{\mathrm{CreER/+}}$*:R26*$^{\mathrm{iDTR/+}}$ mice, *n* = 9; CCR2 antagonist treated C57BL/6 mice, *n* = 7; CCR2-siRNA treated C57BL/6 mice, *n* = 10. IN, infundibulum; Epi, epidermis; HG, hair germ; SG, sebaceous gland; DP, dermal papilla; IF, immunofluorescence analysis; TM, tamoxifen; DT, diphtheria toxin; WT, wild-type. Scale bars, 50 μm.

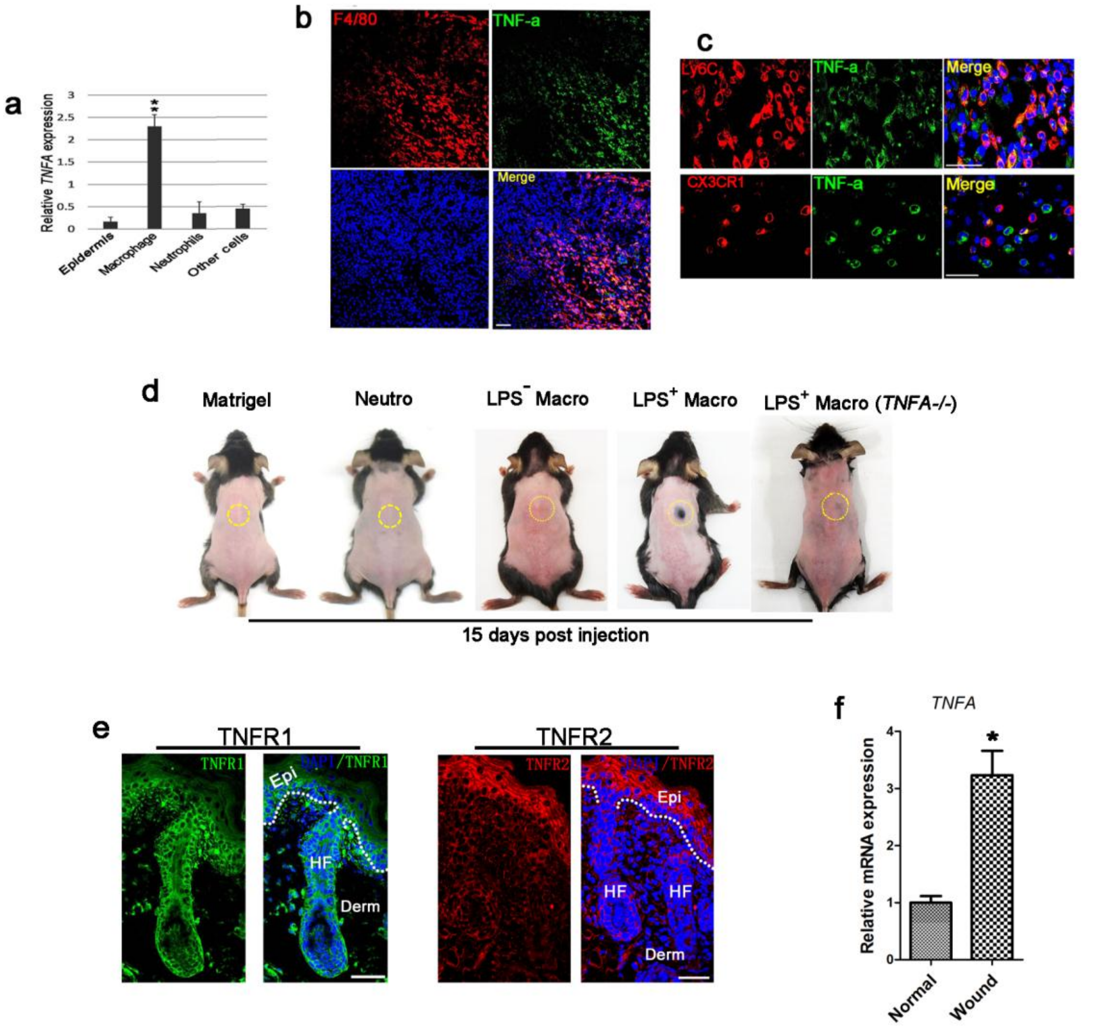

Supplementary Figure 4

(a-c) Real-time PCR analysis of cells derived from wounds (PWD-3) showed much higher *TNFA* expression in macrophages than in epidermal cells, neutrophils and the rest of the cells (Other cells). (b, c) Double staining of wound tissue (PWD-3) indicated that TNF was closely co-localized to F4/80$^+$ macrophages (b) and Ly6C$^+$ macrophages, but barely to CX3CR1$^+$ cells (c). Scale bars, 50 μm.

(d) LPS-activated macrophages induced HF TAT, while LPS-activated macrophages with *TNFA* knockout failed to induce HF TAT. *n*=6 mice for each group.

(e) IF analysis showed that TNFR1 was relatively more highly expressed in the hair follicle and the basal layer of the epidermis, and TNFR2 showed higher expression in the upper layer of the epidermis.

(f) Real-time PCR results showed that in the Tg-TNF mice, the mRNA of TNF in wound-adjacent tissue (PWD-3) was approximately 3-fold higher than that in the non-wounded skin. Data are expressed as the mean ± s.e.m.* $P<0.05$, ** $P<0.01$, unpaired t-test, two-tailed.

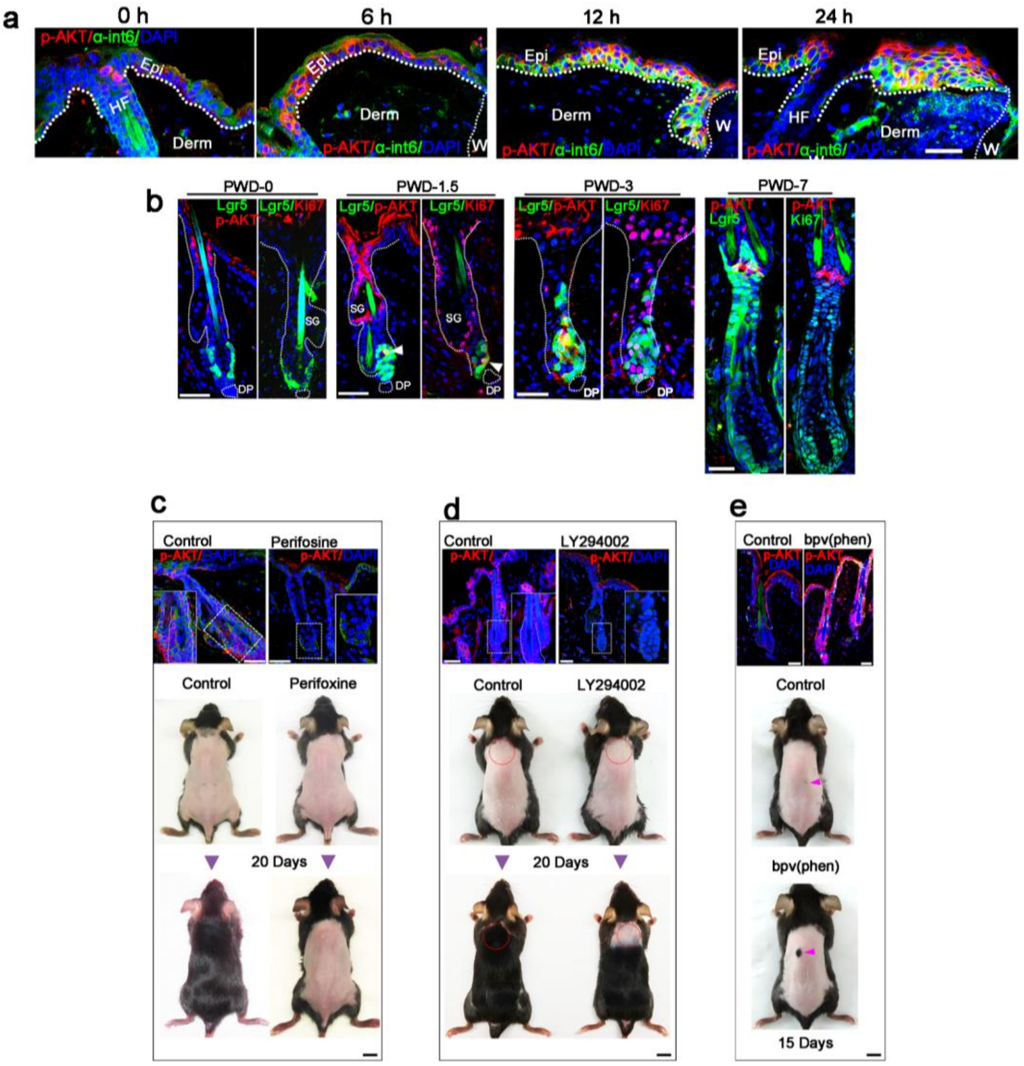

Supplementary Figure 5

(a, b) IF staining indicated that p-AKT was barely detected in the epidermis and HF of unwounded skin. Six hours (h) post-wounding, p-AKT was detected in cells in the basal layer of the epidermis adjacent to the wound, which were also strongly positive for integrin-α6, and increased levels of p-AKT were found at 12 and 24 hours. Five mice for each time point, 2 excisional wounds per mouse, and 5-6 tissue sections per wound were analyzed.

(c-e) Treatment of 8-week-old C57/B6 mice with perifosine (c) or LY294002 (d) suppressed hair depilation-induced p-AKT in epidermal and follicle cells and HF TAT. Control mice, n = 9; perifosine-treated mice, n = 8; LY294002-treated mice, n = 8. Local injection of bpV (phen) into the skin without depilation induced p-AKT in epidermal and HF cells and HF TAT at the injection site (e). n = 8 for both control mice and mice treated with bpV. Scale bars, 50 μm.

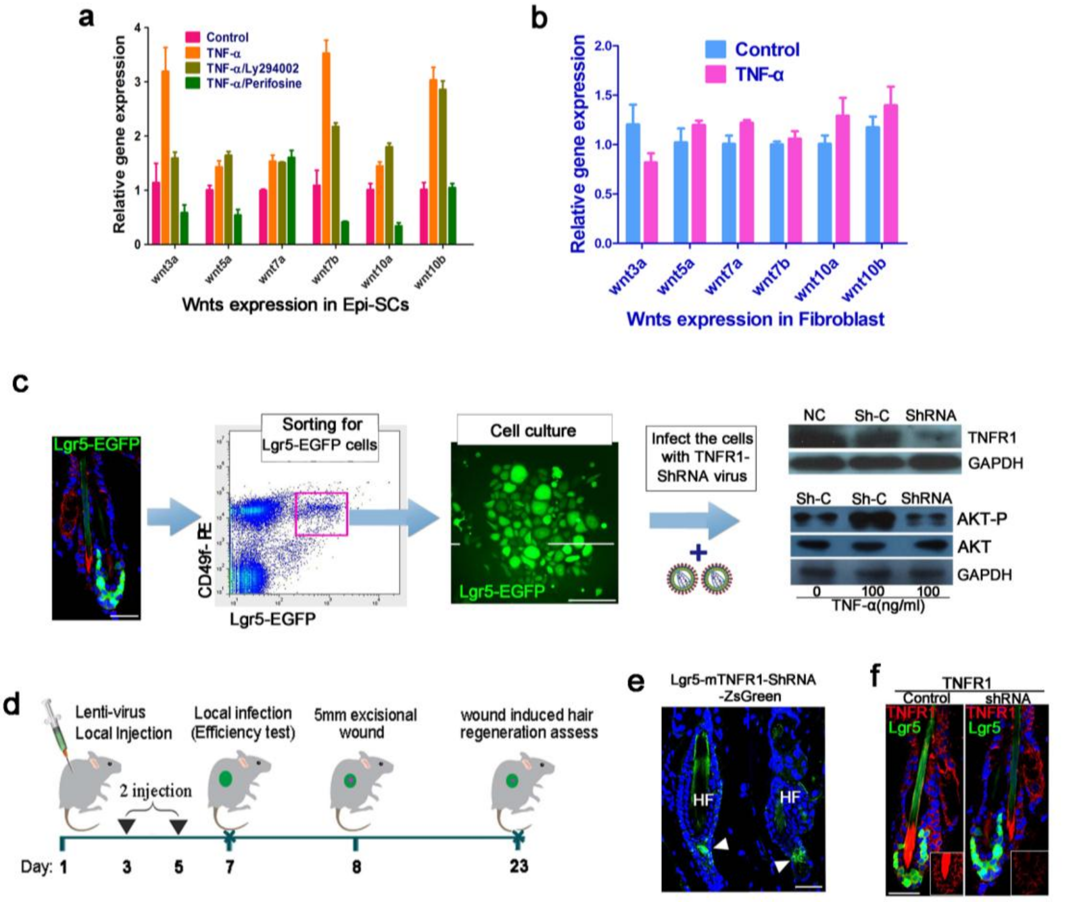

Supplementary Figure 6.

(a) TNF-α markedly increased the expression of Wnt3a, Wnt7b and Wnt10b, but not that of Wnt5a, Wnt7a and Wnt10a, and blockade of PI3K with LY294002 or blockade of AKT with perifosine greatly attenuated TNF-α-induced Wnt ligand expression.

(b) TNF-α exerts a weak effect on the expression of Wnt ligands in fibroblasts.

(c) The efficiency of Lgr5-mTNFR-ShRNA (ShRNA) lentiviruses in the down-regulation of TNFR1 was examined in Lgr5^{+} follicle stem cells isolated from *Lgr5-EGFP* mice by cell sorting for EGFP and CD49f double positive cells, and a mock ShRNA (Sh-C) and culture medium alone (NC) were used as controls; the levels of TNFR1 expression and p-AKT after TNF-α treatment were determined by Western blot analysis, which was repeated 4 times.

(d) A schematic illustration of the experimental scheme of the *in vivo* knockdown of *TNFR1* using ShRNA lentiviruses and its influence on wounding-induced HF TAT.

(e, f) IF analysis of tissue sections of the skin at the injection site. ShRNA-TNFR1 detected the expression of ShRNA (green) in Lgr5^{+} cells (e). The expression of TNFR1 in Lgr5^{+} cells in the HF was substantially down-regulated in mice that received ShRNA-TNFR1 compared to mice receiving the mock sequence (f). Scale bars, 50 μm.

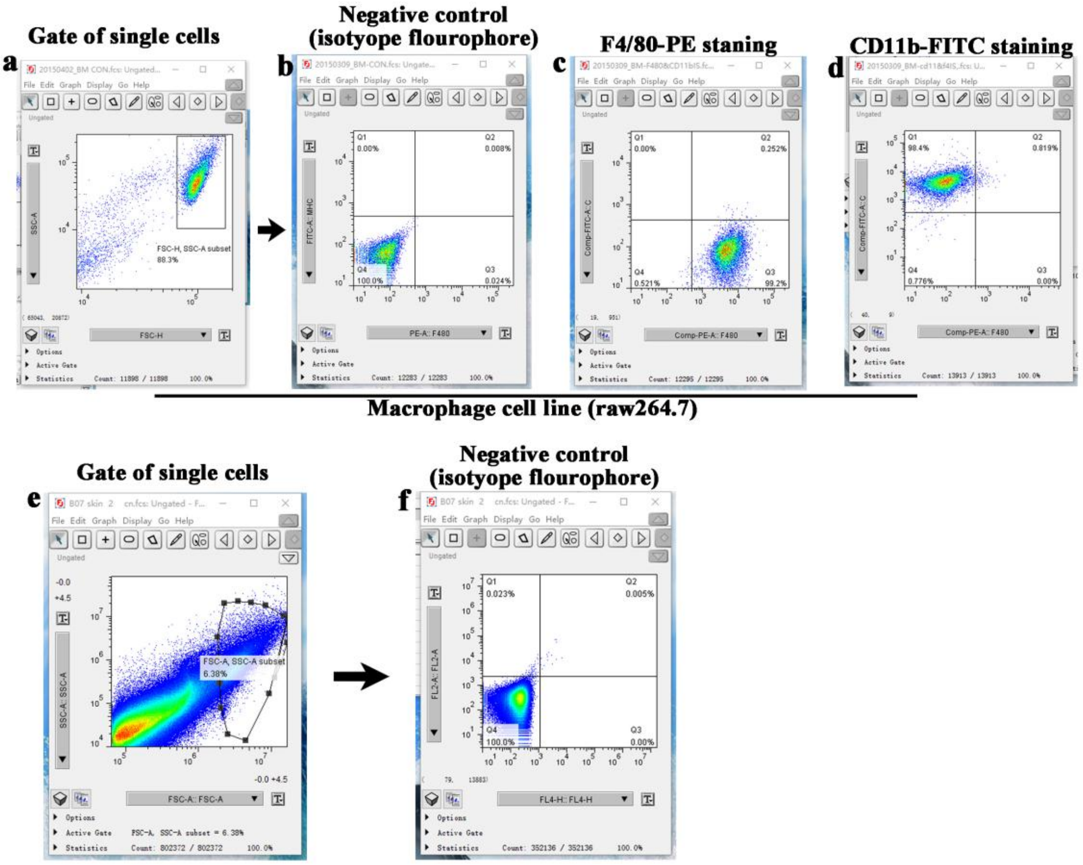

Supplementary Figure 7. Gating strategy for FACS analysis of macrophages in wound tissue (Fig.1c). Firstly, we tested the quality and specificity of the antibodyies (anti-CD11b and F4/80) with a macrophage cell line, set as a positive control. In the analysis, plot was firstly gated for the single cell population (FSC-H vs. SSCA), to remove the debris, air bubbles and laser noise (all which should be FSC-low) (a). The single cell population was further analyzed for their expression of CD11b (FITC) and F4/80 (PE) (c,d); cells stained with FITC/PE Rat IgG2b isotype antibodies were stained as a negative control (b). In the analysis of skin single cell suspensions, plot was also firstly gated for the single cell population (FSC-A vs. SSCA) (e), then the cells were analyzed for the expression of CD11b (FITC) and F4/80 (PE). Similarly, FITC/PE Rat IgG2b isotype antibodies were stained as a negative control, and thus the CD11b$^-$ and F4/80$^-$ cells were gated accordingly (f). With the above parameter of negative and positive controls, cells positive for CD11b and F4/80 in Figure 1c were gated in the plot.

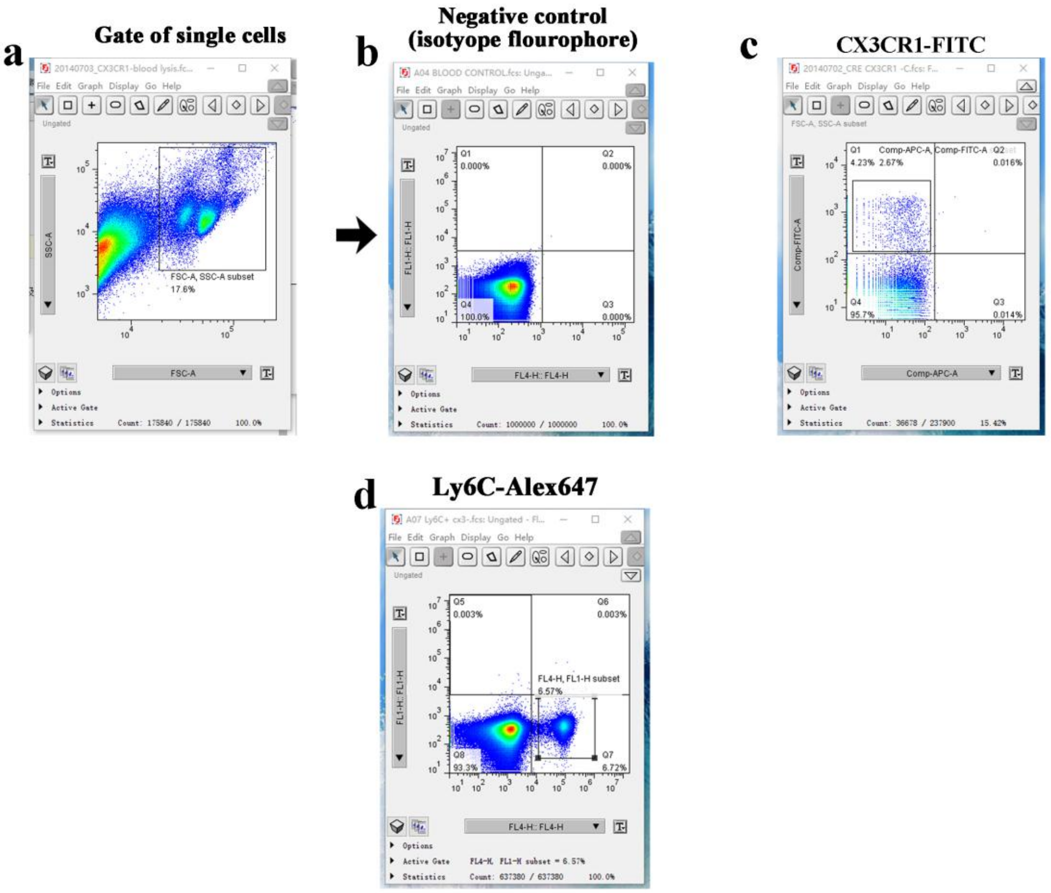

Supplementary Figure 8. Gating strategy for FACS analysis of macrophage sub-populations in blood (Figure 1h and i in main manuscript). In FACS analysis of blood macrophages, plot was firstly gated for single cells (wild type mice) (FSC-A vs. SSCA) (a), then the single cells were analyzed for the expression of CX3CR1 (FITC) (c) and Ly6C (Alexa Fluor 647) (d); Alexa Fluor 647 Rat IgG2b/FITC Mouse IgG2a, κ isotype antibodies stained cells served as a negative control (b). The blood lysis of *CX3CR1CreER-YFP* mice was also analyzed, to detect the sub-population of $CX3CR1^{+}$ cells. With the parameters of negative control and single stains based compensation, cells positive for CX3CR1 or Ly6C were gated in Figure 1h and i.

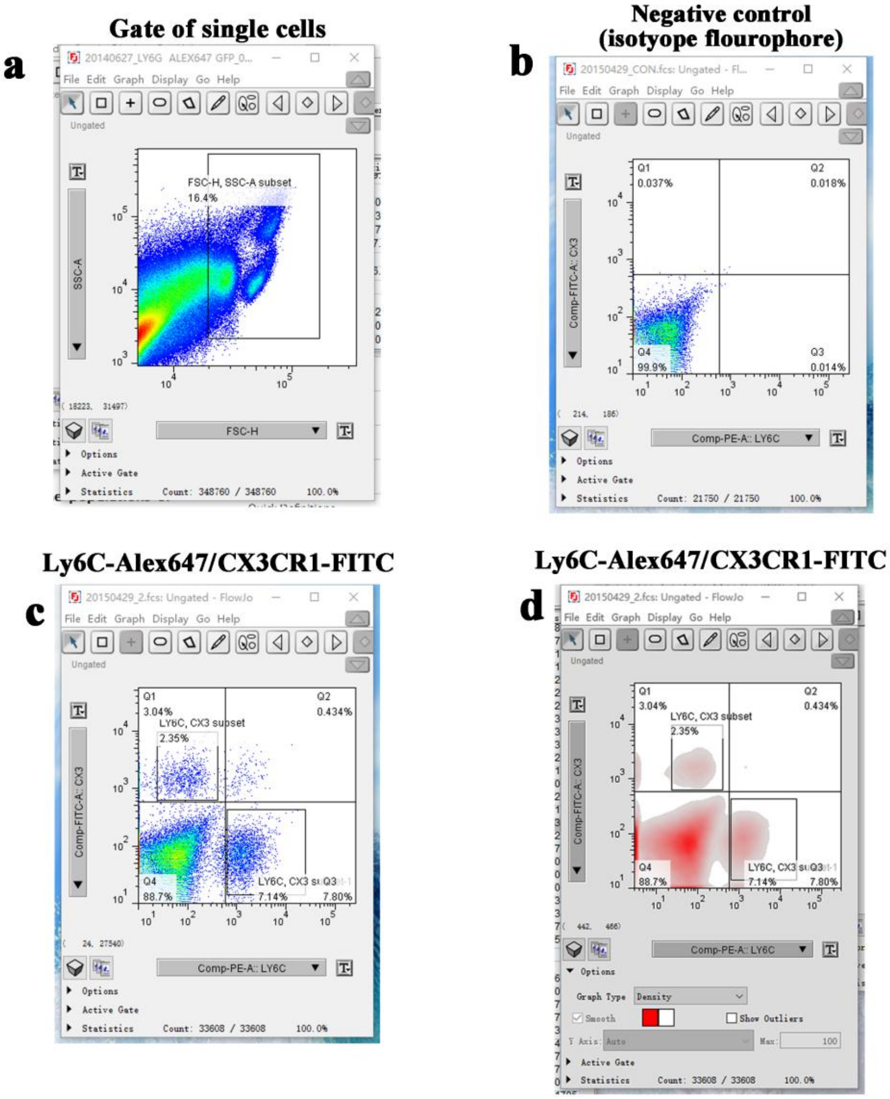

Supplementary Figure 9. Gating strategy for FACS analysis of macrophage sub-populations in blood (Supplementary Figure 3d). In FACS analysis of blood macrophages, plot was firstly gated for single cells (wild type mice) (FSC-A vs. SSCA) (a), and the cells were analyzed for the expression of CX3CR1 (FITC) and Ly6C (Alexa Flour 647); Alexa Fluor 647 Rat rat IgG2b/FITC Mouse IgG2a, κ isotype antibodies stained cells served as a negative control (b). With the gating parameters of negative control, and single staining based compensation, gates for CX3CR1+or Ly6C+ cells were gated (c). The gating strategy was used in Supplementary Figure 3d. In (d) and Supplementary figure 3d, the graph type was present in “Density” style.

Protein size determined based on the visualized protein standards

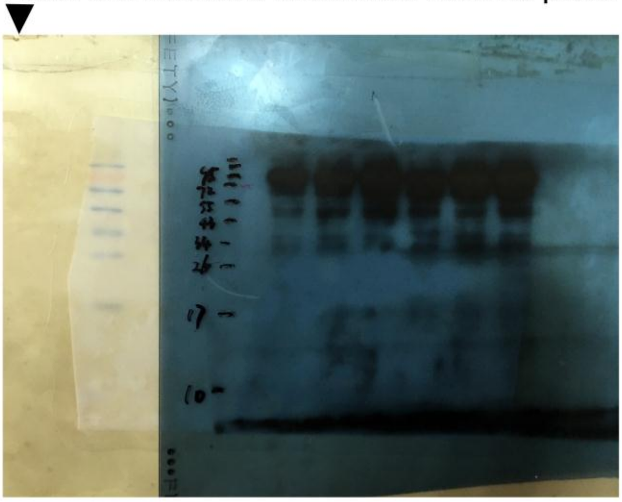

AKT western blot scan

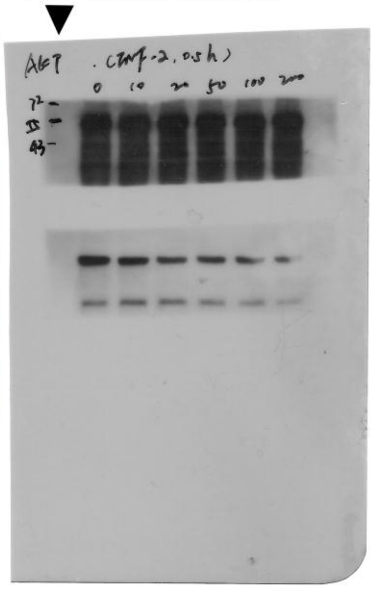

p-AKT western blot scan

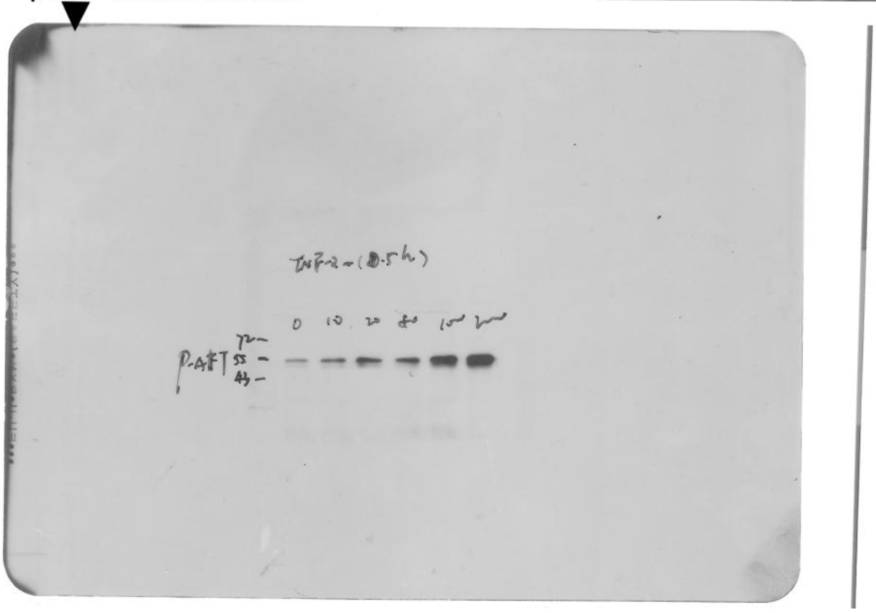

Supplementary Figure 10. Uncropped Western blot scans of Figure 4b in the manuscript.

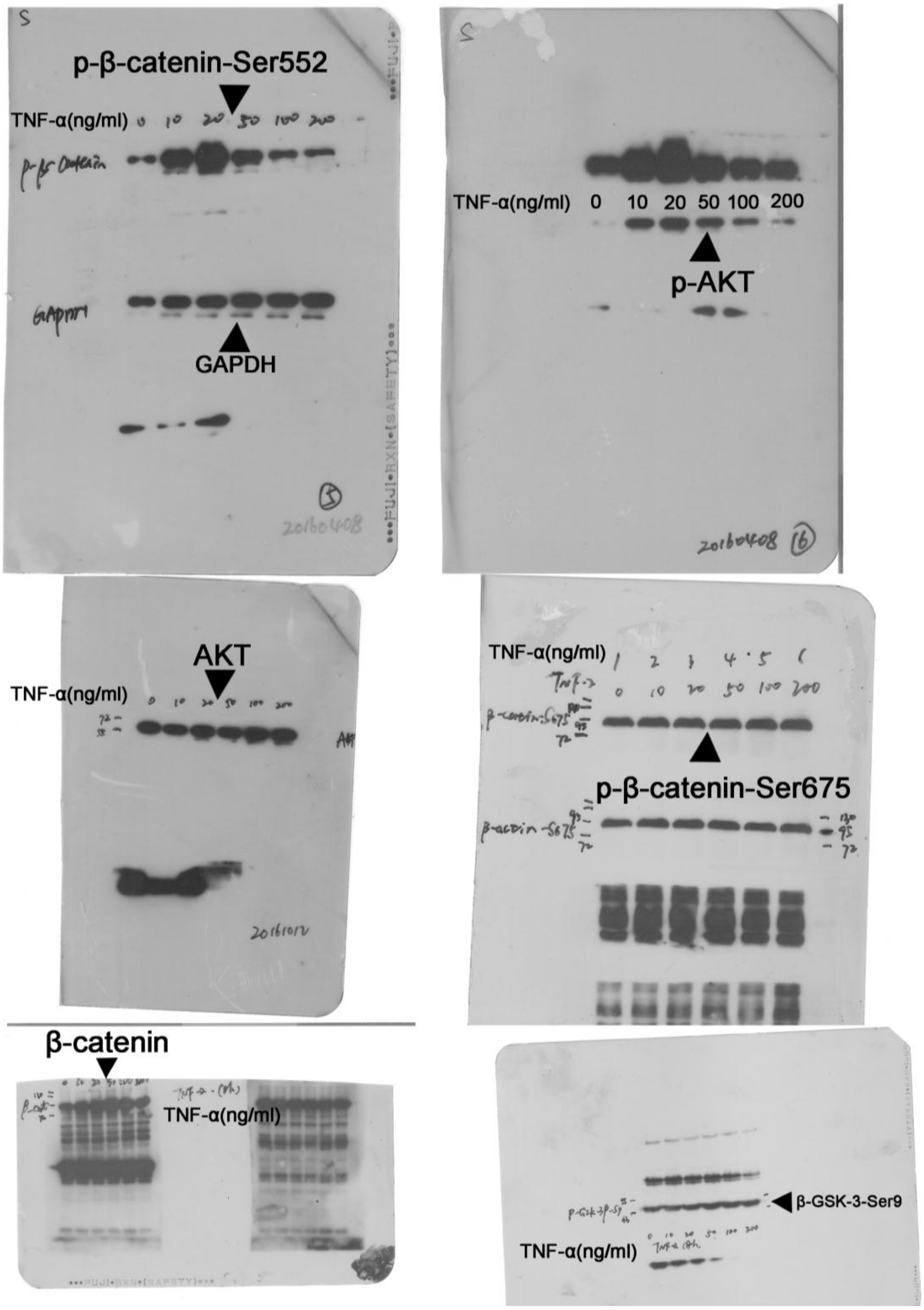

Supplementary Figure 11. Uncropped Western blot scans of Figure 4c in the manuscript.

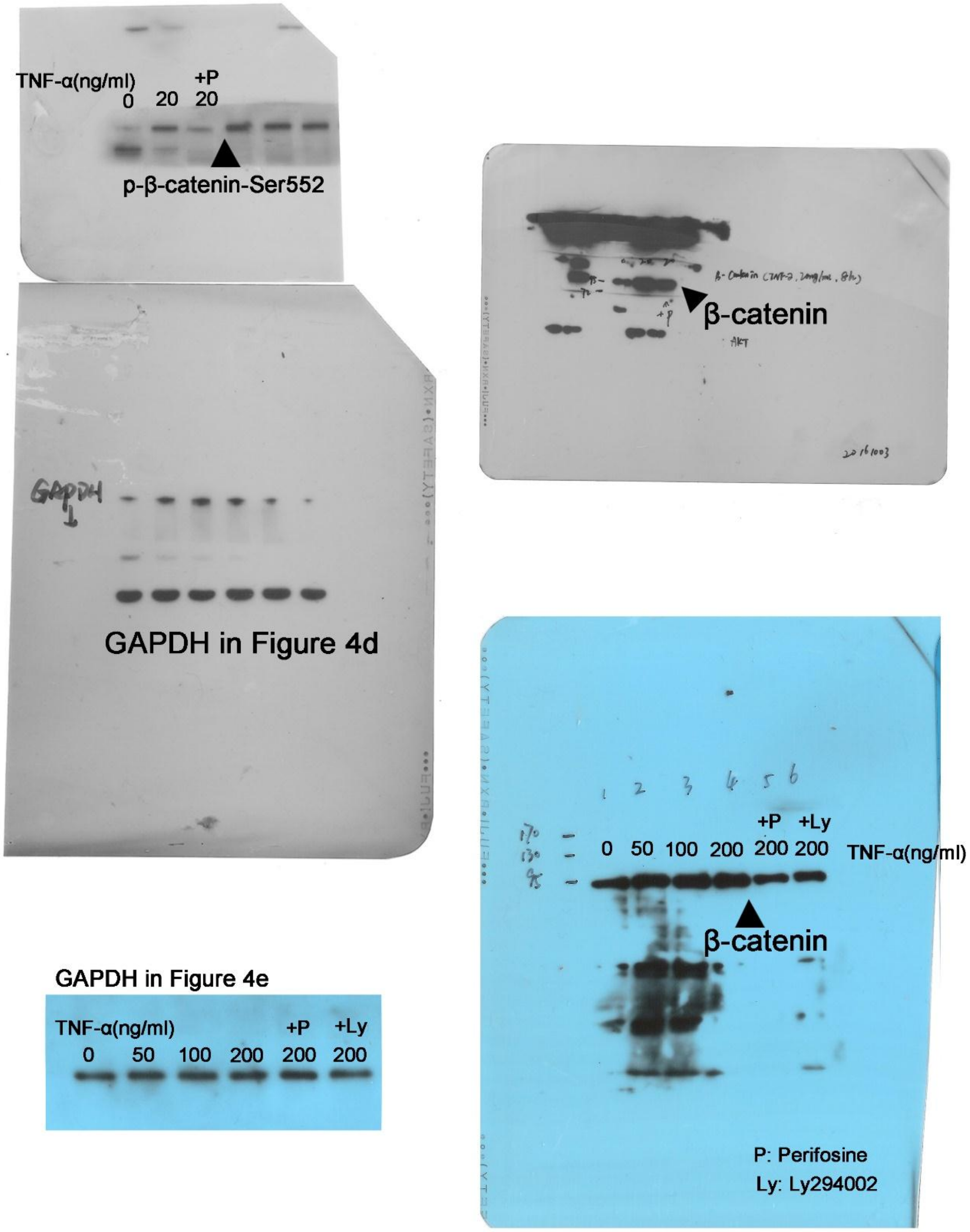

Supplementary Figure 12. Uncropped Western blot scans of Figure 4d and e in the manuscript.

Supplementary Table 1. Genes differentially expressed in Ly6C$^+$ inflammatory macrophages versus CX3CR1$^+$ resident macrophages

	Down-regulation	Up-regulation
Cytokine-cytokine receptor interaction	Bmp2 (-16.5x), Ccl6 (-15.4x), Cd27 (-18.0x), Cxcl13 (-6.9x), Cxcl9 (-2.3x), Cxcr4 (-5.5x), Cxcr5 (-6.1x), Cxcr6 (-53.0x), Epor (-5.8x), Fasl (-3.2x), Flt3 (-2.2x), Ifng (-3.2x), Il12a (-15.2x), Il2 (-7.3x), Il21r (-3.4x), Il2ra (-2.0x), Il2rb (-145.5x), Il4 (-3.0x), Mpl (-36.3x), Ngfr(-9.4x), Tnfrsf13c (-15.0x), Tnfrsf1b (-2.1x), Tnfrsf21 (-10.3x), Tnfrsf25 (-13.6x),	Amh (2.1x), Ccl4 (2.6x), Csf2 (8.1x), Csf3 (30.2x), Cxcl1 (3.1x), Cxcl10 (109.5x), Cxcl2 (13.2x), Egfr (7.0x), Ifnb1 (12.1x), Il10 (2.7x), Il10ra (2.3x), Il1a (21.2x), Il23a (17.2x), Il6 (7.9x), Lif (14.3x), Osm (5.3x), Pdgfb (2.7x), Tnf (11.8x), Tnfrsf12a (2.7x), Tnfsf9 (3.3x), Vegfa (3.0x), Cxcl16 (1.2x)
T cell receptor signaling pathway	Akt3 (-199.2x), Cd28 (-15.5x), Cd3d (-5.5x), Cd3e (-69.3x), Cd3g(-57.2x), Cd8a (-28.5x), Cd8b1 (-61.6x), Ctla4 (-3.1x), Grap2 (-965.4x), Ifng (-3.2x), Il2 (-7.3x),	Csf2 (8.1x), Fos (9.1x), Il10 (2.7x), Jun (2.7x), Tnf (11.8x)
Natural killer cell mediated cytotoxicity	Fasl (-3.2x), Gzmb (-33.5x), Ifng (-3.2x), Itgb2l (-19.6x), Klra1 (-3.5x), Klra9 (-4.6x), Klrb1c (-22.3x), Klrc1 (-12.8x), Lat (-2.2x), Ncr1 (-2.8x), Nfat5 (-2.3x), Prkcb (-38.3x), Sh2d1a (-55.6x), Shc2 (-2.7x),	Csf2 (8.1x), Icam1 (5.7x), Ifnb1 (12.1x), Tnf (11.8x)
Wnt signaling pathway	Cxxc4 (-2.3x), Fzd2 (-23.3x), Nfat5 (-2.3x), Plcb1 (-2.4x), Ppp2r2(-2.1x), Prkcb (-38.3x), Sox17 (-12.1x), Tcf7 (-9.8x), Vangl1 (-22.1x), Wnt16 (-2.5x), Wnt8b (-2.6x)	Camk2b (1.3x), Ccnd2 (1.3x), Fosl1 (3.0x), Fzd4 (2.5x), Jun (2.7x), Myc (5.2x), Prkx (2.3x), Skp1a (4.8x), Wnt3 (2.0x)
TGF-beta signaling pathway	Bmp2 (-16.5x), Bmp4 (-2.0x), Bmpr1b (-2.7x), Chrd (-2.3x), Fst (-2.3x), Ifng (-3.2x), Ltbp1 (-13.1x), Pitx2 (-13.0x), Ppp2r2c (-2.1x)	Amh (2.1x), Id3 (3.5x), Myc (5.2x), Skp1a (4.8x), Smad5 (2.2x), Tnf (11.8x)
Cell Communication	4732456N10Rik (-21.1x), Col1a2 (-3.8x), Dsc3 (-5.9x), Dsg4 (-2.5x), Gja5 (-2.4x), Krt12 (-2.1x), Krt15 (-3.2x), Krt17 (-6.9x), Krt27 (-2.3x),	Krt20 (3.3x), Lamc2 (4.5x)
VEGF signaling pathway	Akt3 (-199.2x), Mapkapk3 (-7.2x), Nfat5 (-2.3x), Pla2g10 (-5.9x), Pla2g12b (-2.1x), Pla2g2c (-2.9x),	Ptgs2 (2.3x), Vegfa (3.0x)
Toll-like receptor signaling pathway	Akt3 (-199.2x), Cxcl9 (-2.3x), Il12a (-15.2x)	Ccl4 (2.6x), Cxcl10 (109.5x), Fos (9.1x), Ifnb1 (12.1x), Ikbke (2.6x), Il6 (7.9x), Jun (2.7x), Tirap (1.2x),

Supplementary Table 2. Primers for Real-Time PCR analysis

**Genes**	**Forward**	**Reverse**
CCL4	TTCCTGCTGTTTCTCTTACACCT	CTGTCTGCCTCTTTTGGTCAG
IL6	CTGCAAGAGACTTCCATCCAG	AGTGGTATAGACAGGTCTGTTGG
IL10	CTTACTGACTGGCATGAGGATCA	GCAGCTCTAGGAGCATGTGG
PDGFB	AGGAGTGATACCAGCTTTAGTCC	CCGAGCAGGTCAGAACAAAGG
IL2	TGAGCAGGATGGAGAATTACAGG	GTCCAAGTTCATCTTCTAGGCAC
TNFA	CAGGCGGTGCCTATGTCTC	CGATCACCCCGAAGTTCAGTAG
IL1b	GAAATGCCACCTTTTGACAGTG	TGGATGCTCTCATCAGGACAG
WNT7A	CCCGCCACCAGCGGGGACTA	TGCTCGCACCCAGAGCTACCACC
FSCN1	GACGAGATCGCGGTAGACC	AGGTAGCGACCTTCGCAGT
BMP6	AGCGCTTTCCTCAACGACGCGG	AGCCGTCACCGCCTCACCCT
CSF1	GTGTCAGAACACTGTAGCCAC	TCAAAGGCAATCTGGCATGAAG
BMP2	AGCCCAGTTGCTGCTCCAGGTCC	AAGACAGCGGGTCCCGGCCA
FASLG	CAGCCCATGAATTACCCATGT	ATTTGTGTTGTGGTCCTTCTTCT
FLT3	CTTGAGACCGTTACAAACCAAGA	CATTCGGTACGATGATGGCTTT
PPBP	CTCAGACCTACATCGTCCTGC	GTGGCTATCACTTCCACATCAG
WNT10B	CCAAGAGCCGGGCCCGAGTGA	AAGGGCGGAGGCCGAGACCG
GAPDH	TGGGGTGAGGCCGGTGCTGA	GGCATCGGCAGAAGGGGCGG
